# Atomistic Modeling of Quaternized Chitosan Head Groups: Insights into Chemical Stability and Ion Transport for Anion Exchange Membrane Applications

**DOI:** 10.3390/molecules29133175

**Published:** 2024-07-03

**Authors:** Mirat Karibayev, Bauyrzhan Myrzakhmetov, Dias Bekeshov, Yanwei Wang, Almagul Mentbayeva

**Affiliations:** 1 Department of Chemical & Materials Engineering, School of Engineering and Digital Sciences, Nazarbayev University, Astana 010000, Kazakhstan; mirat.karibayev@nu.edu.kz (M.K.); dias.bekeshov@nu.edu.kz (D.B.); 2 Center for Energy and Advanced Materials Science, National Laboratory Astana, Nazarbayev University, Astana 010000, Kazakhstan; bauyrzhan.myrzakhmetov@nu.edu.kz

**Keywords:** anion exchange membrane, quaternized chitosan, chemical stability, density functional theory, molecular dynamics, hydroxide ion transport

## Abstract

The chemical stability and ion transport properties of quaternized chitosan (QCS)-based anion exchange membranes (AEMs) were explored using Density Functional Theory (DFT) calculations and all-atom molecular dynamics (MD) simulations. DFT calculations of LUMO energies, reaction energies, and activation energies revealed an increasing stability trend among the head groups: propyl trimethyl ammonium chitosan (C) < oxy propyl trimethyl ammonium chitosan (B) < 2-hydroxy propyl trimethyl ammonium chitosan (A) at hydration levels (HLs) of 0 and 3. Subsequently, all-atom MD simulations evaluated the diffusion of hydroxide ions (${\mathrm{OH}}^{-}$) through mean square displacement (MSD) versus time curves. The diffusion coefficients of ${\mathrm{OH}}^{-}$ ions for the three types of QCS (A, B, and C) were observed to increase monotonically with HLs ranging from 3 to 15 and temperatures from 298 K to 350 K. Across different HLs and temperatures, the three QCS variants exhibited comparable diffusion coefficients, underlining their effectiveness in vehicular transport of ${\mathrm{OH}}^{-}$ ions.

## 1. Introduction

Anion exchange membrane fuel cells (AEMFCs) have gained prominence because of their potential for high efficiency and cost-effectiveness in energy conversion [1,2,3,4]. These systems utilize solid polymeric electrolytes and operate at lower temperatures compared to other types of fuel cells. Among various materials explored for AEMFCs, polysaccharide-based biopolymers, particularly chitosan, have attracted significant attention. Chitosan, a natural biopolymer derived from chitin, is abundant and environmentally benign, offering an attractive alternative to traditional petroleum-derived polymers [5].

The inherent structural properties of chitosan, including its film-forming ability and mechanical strength, render it an exceptional candidate for the development of anion exchange membranes (AEMs). Furthermore, enhancing the ionic conductivity of chitosan through quaternization makes it particularly suitable for high-performance AEMFCs [6,7,8,9]. Despite these advantages, the detailed molecular mechanisms involved in the transport of ions and the chemical stability of quaternized chitosan (QCS) under operational conditions remain underexplored. This is particularly crucial at lower hydration levels (HLs), where ionic transport and membrane stability are challenging [10,11].

In this context, our study employs Density Functional Theory (DFT) and classical all-atom molecular dynamics (MD) simulations to explore the chemical stability and ion transport properties of QCS-based AEMs. We focus on the interaction dynamics of hydroxide ions (${\mathrm{OH}}^{-}$) within the polymer matrix and investigate the influence of different levels of hydration and temperatures on the performance of QCS-based AEMs. Through computational modeling, this study aims to provide a deeper understanding of the physicochemical interactions at play, which are crucial to optimizing the design of AEMs for sustainable energy applications.

Our approach includes an analysis of the lowest unoccupied molecular orbital (LUMO) energies, reaction energies ($\Delta E_{\mathrm{reaction}}$), and activation energies ($\Delta E_{\mathrm{activation}}$), alongside evaluations of diffusion coefficients using mean square displacement (MSD) vs. time curves. This comprehensive investigation elucidates the fundamental mechanisms that underpin the transport and stability characteristics of quaternized chitosan in AEM applications, contributing to the field of renewable energy technologies.

## 2. Results and Discussion

### 2.1. DFT Results

DFT-based quantum chemical calculations provide valuable insights into the chemical stability and quantum chemical features of various QCS derivatives. This section focuses on $\Delta E_{\mathrm{reaction}}$ and $\Delta E_{\mathrm{activation}}$ for the $S_{N}2$ degradation processes, as well as on the distribution and energies of LUMO.

#### 2.1.1. Degradation Reactions

The operating environment of AEMFCs can lead to dry conditions at the cathode electrode due to the increased current density, resulting in a lower water content and enhanced ${\mathrm{OH}}^{-}$ ion formation. This condition facilitates the nucleophilic attack of ${\mathrm{OH}}^{-}$ ions on the QA head groups of QCS via $S_{N}2$ degradation processes, particularly in high pH conditions [12,13]. At high pH, ${\mathrm{OH}}^{-}$ ions become more nucleophilic, and this enhanced nucleophilicity can lead to the degradation of QA head groups in AEM. [12,13]. The B3LYP DFT method was used to further assess the chemical degradation reaction between the ${\mathrm{OH}}^{-}$ ion and QCS, as well as to calculate the $\Delta E_{\mathrm{reaction}}$, $\Delta E_{\mathrm{activation}}$, and transition state for different QCS. Here, ${\mathrm{OH}}^{-}$ ions have the potential to target the methylene carbon atoms of QCS, which could lead to chemical degradation via the $S_{N}2$ degradation process.

**Figure 1:**
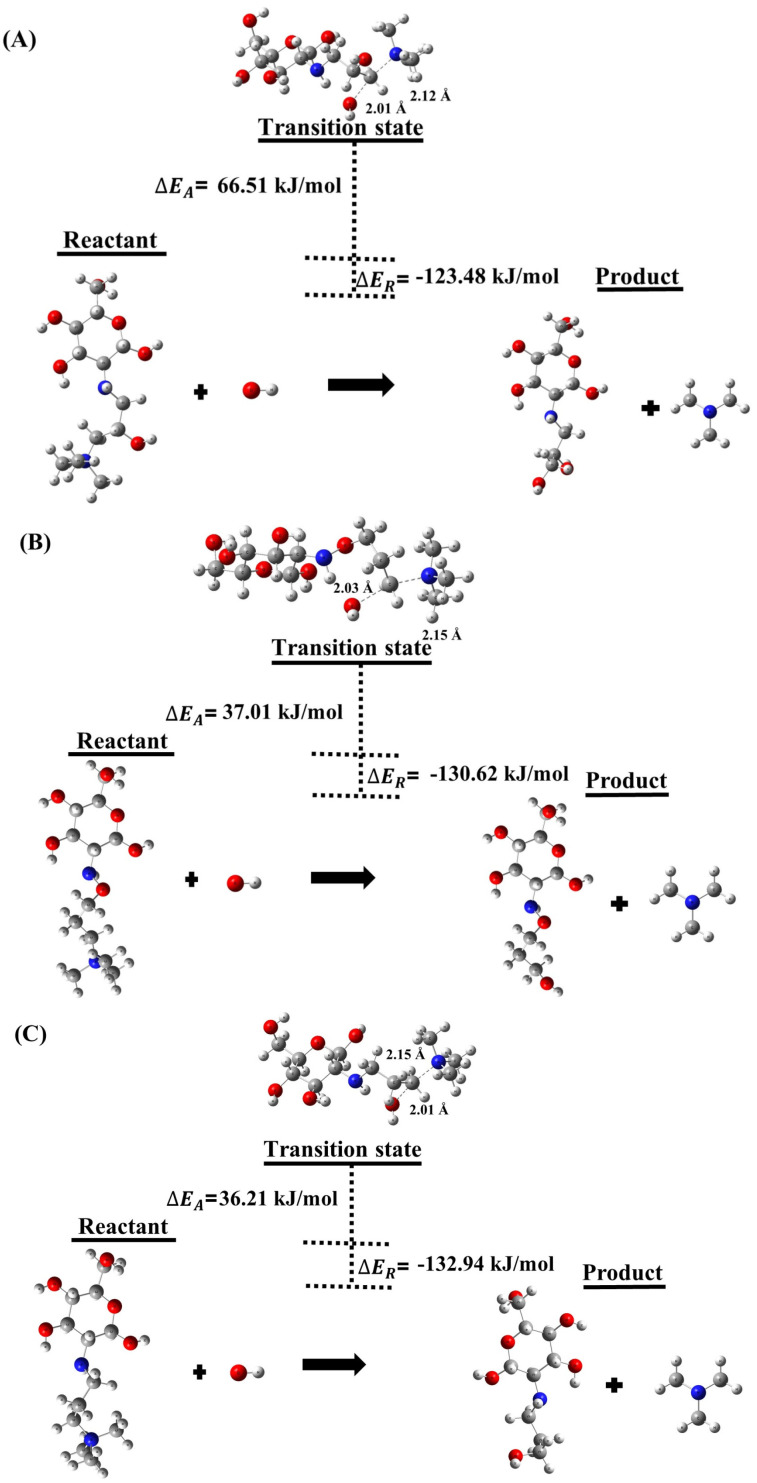
Depiction of $S_{N}2$ degradation reactions for QCS segments (**A**–**C**) at the HL 0.

**Figure 2:**
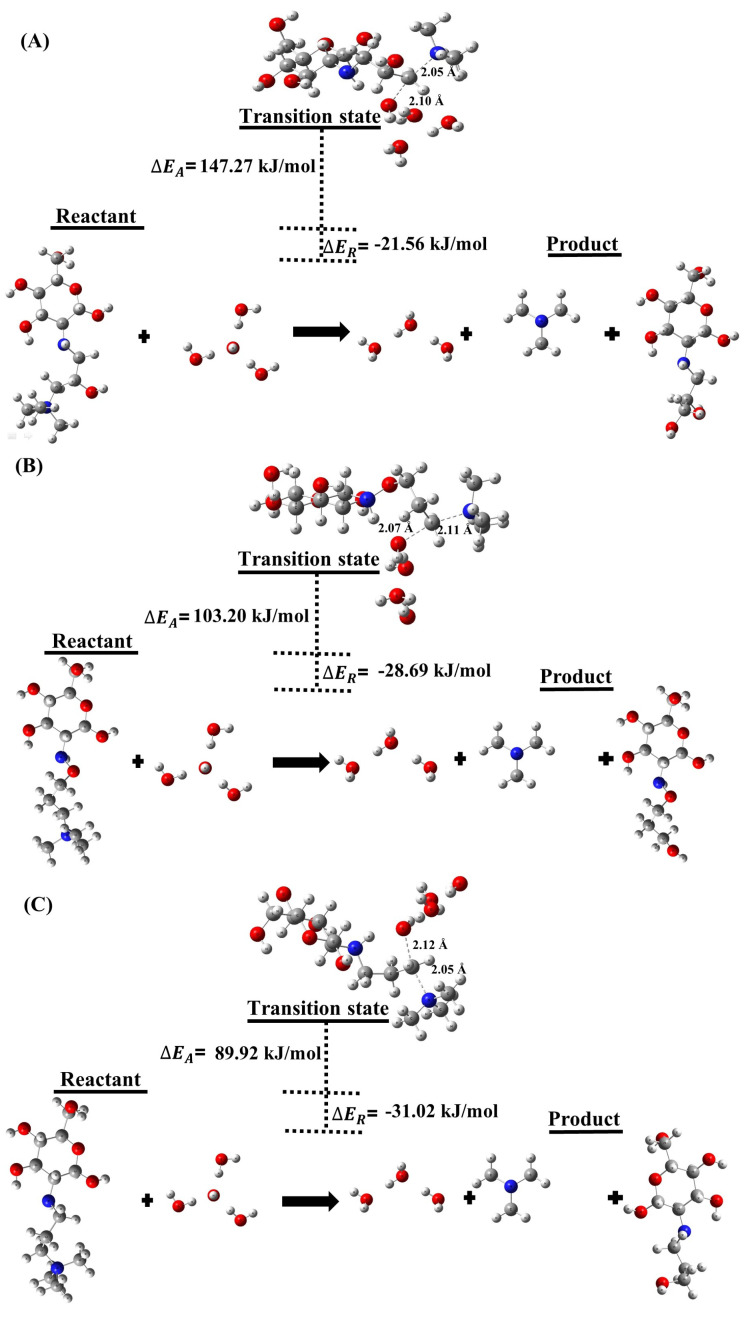
Depiction of $S_{N}2$ degradation reactions for QCS segments (**A**–**C**) at the HL 3.

Table 1 and Table 2 as well as Figure 1 and Figure 2 present our results of $\Delta E_{\mathrm{reaction}}$, transition state, and $\Delta E_{\mathrm{activation}}$ for the degradation $S_{N}2$ of the different QCS in alkaline media in the HL of 0 and 3. The $S_{N}2$ degradation mechanism involves the attack of a nucleophile, such as ${\mathrm{OH}}^{-}$ ion, on the QCS, leading to bond cleavage and degradation of the AEM. Our overall DFT transition state calculations reveal that the $\Delta E_{\mathrm{reaction}}$ for $S_{N}2$ degradation of the QCS is −123.48kJ/mol (HL 0), and −21.56kJ/mol (HL 3) for (A), −130.62kJ/mol (HL 0) and −28.69kJ/mol (HL 3) for (B), and, −132.94kJ/mol (HL 0) and −31.02kJ/mol (HL 3) for (C) as shown in Figure 1 and Figure 2. The results have significant implications for the stability and durability of AEMs. In particular, our DFT calculations indicate that the overall reaction associated with QCS (A) exhibits a less exothermic $\Delta E_{\mathrm{reaction}}$ compared to the values observed for QCS (B) and QCS (C). This suggests that AEMs containing QCS (B) and QCS (C) are more prone to degradation when exposed to nucleophiles, such as ${\mathrm{OH}}^{-}$ ions, in contrast to AEMs incorporating QCS (A).

Figure 1 and Figure 2 also show the $\Delta E_{\mathrm{activation}}$ for ${\mathrm{OH}}^{-}$ ion attack on the QCS is 66.51 kJ/mol (HL 0) and 147.27 kJ/mol (HL 3) for (A), 37.01 kJ/mol (HL 0) and 103.20 kJ/mol (HL 3) for (B), and 36.21 kJ/mol (HL 0) and 89.92 kJ/mol (HL 3) for (C). It reveals that QCS (B) and (C) of the AEM degrade much faster compared to QCS (A), according to this statement. The higher $\Delta E_{\mathrm{activation}}$ for QCS (A) shows that the quaternized head group of chitosan is more resistant to ${\mathrm{OH}}^{-}$ ion attack and therefore more stable compared to QCS (B) and QCS (C). The lower $\Delta E_{\mathrm{activation}}$ values for QCS (B) and (C) reveal that the quaternized head group of chitosan is less stable and can degrade more easily under the attack of ${\mathrm{OH}}^{-}$ ions. A closer examination from a chemical structural perspective reveals that QCS (A) possesses an alkoxy group surrounding a carbon atom, which is susceptible to attack by ${\mathrm{OH}}^{-}$ ions through $S_{N}2$. Interestingly, the presence of this protecting group in QCS (A) hinders ${\mathrm{OH}}^{-}$ ion attachment, an effect not observed in QCS (B) and QCS (C). This hindrance contributes to an increased activation barrier, suggesting improved chemical stability. These insights demonstrate the importance of protective groups in improving the stability of QCS, shedding light on potential strategies for the development of more stable and durable AEMs.

In prior experimental studies, it was observed that the synthesis of QCS was achieved by the use of glycidyltrimethylammonium chloride precursors, resulting in the production of two isomeric forms of QCS, namely QCS (A) and QCS (B). In particular, QCS (A) [8,14,15] has been extensively investigated in the field of anion exchange membrane (AEM) applications compared to the study of QCS (B) and QCS (C) [16]. In our previous work, the degradation mechanism of two different QA head groups was studied by $\Delta E_{\mathrm{reaction}}$ and $\Delta E_{\mathrm{activation}}$ from HL 0 to 3 using DFT calculations [17]. The chemical stability of trimethylhexylammonium was higher than that of benzyltrimethylammonium because $\Delta E_{\mathrm{activation}}$ of benzyltrimethylammonium was lower than $\Delta E_{\mathrm{activation}}$ of trimethylhexylammonium, while $\Delta E_{\mathrm{reaction}}$ for benzyltrimethylammonium and trimethylhexylammonium was similar in the different HLs [17]. Further comparison of our previous work with current work states that the chemical stability of QCS is higher in comparison with benzyltrimethylammonium, and trimethylhexylammonium from our previous work. By optimizing the chemical structure of the QCS, it may be possible to develop AEMs that are more resistant to nucleophilic attack and have better long-term stability and durability.

#### 2.1.2. LUMO Distribution and Energies

The LUMO energies play a pivotal role in understanding the reactivity and stability of QCS variants. In our analysis, the ascending order of the LUMO energies correlates directly with the chemical stability observed in the transition state calculations of $\Delta E_{\mathrm{reaction}}$ and $\Delta E_{\mathrm{activation}}$. A higher LUMO energy (less negative) generally indicates that it is more difficult for the molecule to accept electrons. In the context of nucleophilic attacks, such as those involving ${\mathrm{OH}}^{-}$ ions in this study, a higher LUMO energy means that the molecule is less prone to react with nucleophiles. This is because nucleophiles donate electrons, and a higher LUMO energy implies a lower propensity to accept these electrons.

**Figure 3:**
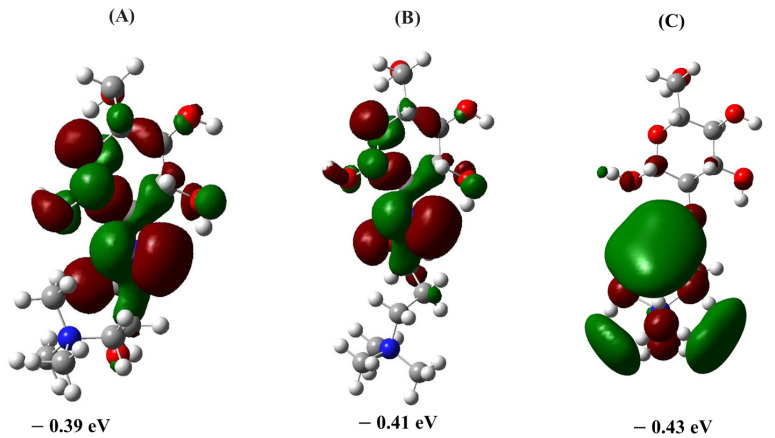
Representation of LUMO densities for the optimized ground state geometries of QCS segments, including (**A**) 2-hydroxy propyl trimethyl ammonium chitosan, (**B**) oxy propyl trimethyl ammonium chitosan, and (**C**) propyl trimethyl ammonium chitosan.

Figure 3 illustrates these LUMO energies, confirming the stability order from QCS (C) to QCS (A). Specifically, QCS (A), which shows the highest LUMO energy among the variants, exhibits the greatest chemical resistance, as demonstrated by its higher $\Delta E_{\mathrm{activation}}$ and less exothermic $\Delta E_{\mathrm{reaction}}$ (see Table 1 and Table 2). Furthermore, QCS (A) displays not only a higher LUMO energy but also a more distributed LUMO within the chitosan moiety, enhancing its protective effect against nucleophilic attacks. This observation is consistent with our previous studies [17], which have established a similar correlation between LUMO energies and chemical stability in different QA head groups, thus strengthening the reliability of using LUMO energy as a predictive tool to evaluate the alkaline stability of QCS in AEM applications [17]. The comparative analysis with previous findings further underscores the value of using LUMO energies as an efficient and less computationally demanding method to gauge the long-term durability and stability of AEM materials.

### 2.2. Classical All-Atom MD Results

#### 2.2.1. Effect of Hydration Level

**Figure 4:**
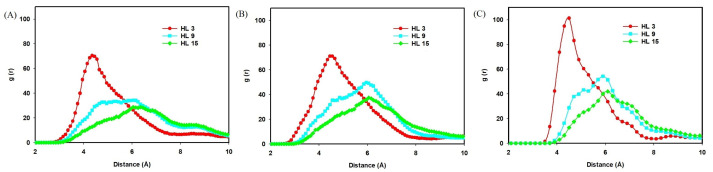
RDFs for the correlation between the oxygen atom of ${\mathrm{OH}}^{-}$ ion and the nitrogen atom of QCS (types **A**–**C**) in the presence of explicit water molecules at the different HLs.

The correlation between the nitrogen atom of QCS and the ${\mathrm{OH}}^{-}$ ion was investigated by computing the radial distribution functions from classical all-atom MD simulations. The RDF profiles (Figure 4) describe the likelihood of finding the oxygen atom of the ${\mathrm{OH}}^{-}$ ion at various distances from the nitrogen atoms of QCS. As shown in Figure 4, the peak values of the RDFs indicate the spatial relationship between the ${\mathrm{OH}}^{-}$ ions and QCS at different hydration levels. At HL 3, the highest peak of 70.4 at 4.4 Åcorresponds to a close approach between the oxygen of ${\mathrm{OH}}^{-}$ and the nitrogen of QCS (A). As the hydration level increases to HL 9 and 15, the peak values decrease to 34.3 at 5.4 Åand 28.7 at 6.2 Å, respectively, indicating a reduction in interaction strength. This trend suggests that increased hydration leads to an expected dilution effect, which reduces the strength of close interactions between ${\mathrm{OH}}^{-}$ ions and QCS.

**Figure 5:**
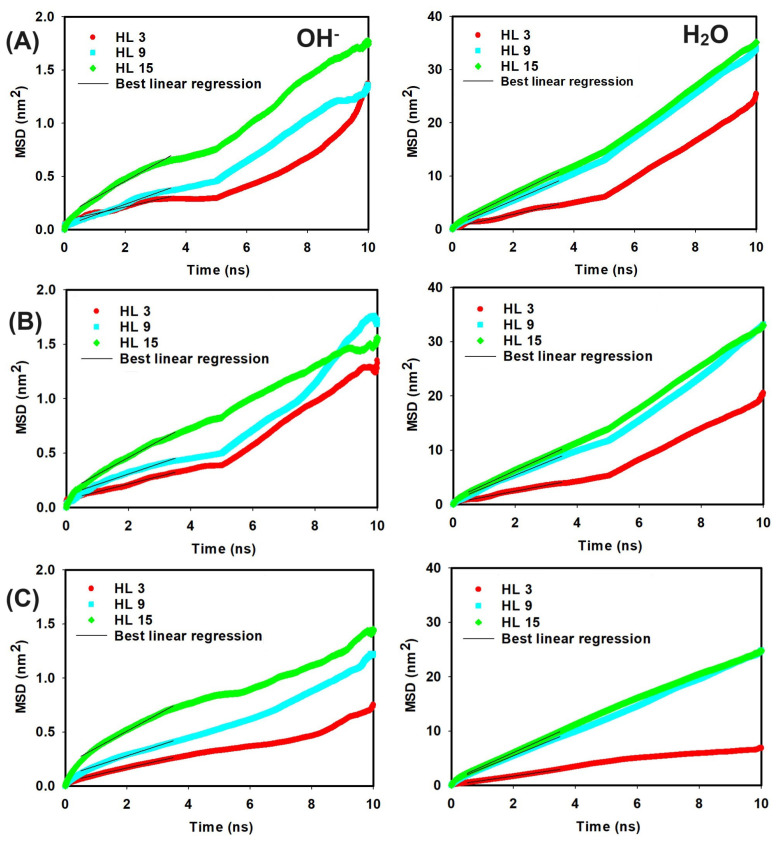
MSD vs. time curves at different HLs for ${\mathrm{OH}}^{-}$ ions and $H_{2}O$ molecules in systems with QCS types (**A**–**C**), respectively.

To investigate the influence of hydration levels on ion mobility, the mean square displacement was analyzed for ${\mathrm{OH}}^{-}$ ions and water molecules in different HLs, as shown in Figure 5. The slopes of the linear region of the resulting MSD vs. time curves were used to calculate the diffusion coefficients, which are tabulated in Table 3 and Table 4. Our results of the diffusion coefficients of ${\mathrm{OH}}^{-}$ ions align closely with those reported in previous studies [18,19]. The diffusion coefficients of ${\mathrm{OH}}^{-}$ ions and water molecules show a clear increase as the hydration level rises from 3 to 15. For instance, in the presence of QCS (A), the diffusion coefficient of ${\mathrm{OH}}^{-}$ ions escalates from 0.011 to 0.027 ${\mathrm{nm}}^{2}/\mathrm{ns}$, while that of water molecules jumps from 0.20 to 0.46 ${\mathrm{nm}}^{2}/\mathrm{ns}$. Similar trends are observed for QCS (B) and QCS (C), underscoring a consistent increase in mobility with a higher water content.

**Figure 6:**
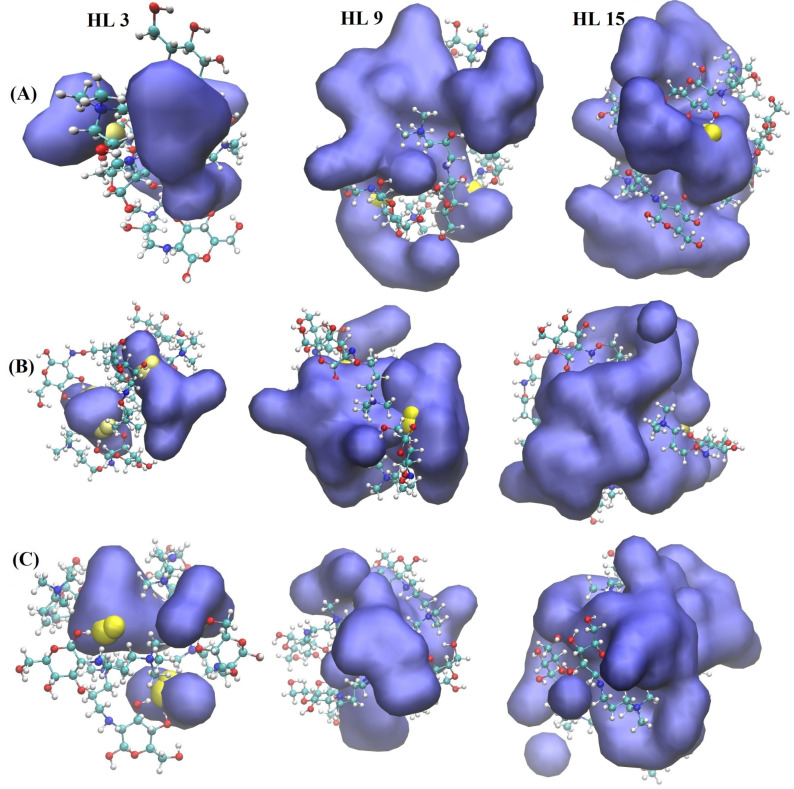
Snapshot of water clusters surrounding QCS types (**A**–**C**) AEMs at various HLs. Drawing method for water: Quick Surface and color scheme for water: blue; Drawing method for ${\mathrm{OH}}^{-}$ ion: VdW representation and color scheme for ${\mathrm{OH}}^{-}$ ion: yellow (oxygen), grey (hydrogen); Drawing method for QCS: CPK representation and color scheme for QCS: violet (carbon), grey (hydrogen), red (oxygen), blue (nitrogen).

Representative snapshots of molecular configurations around QCS groups are presented in Figure 6, which shows the structural configuration of QCS (in the CPK representation), ${\mathrm{OH}}^{-}$ ions (in the VdW representation), and water molecules (in the Quick Surface representation). The results show that water molecules form a bicontinuous network [20,21,22,23] at high HLs. This visualization helps to understand the microstructural dynamics that contributes to the diffusion behavior observed in Figure 5.

In summary, increasing the level of hydration not only enhances the mobility of ${\mathrm{OH}}^{-}$ ions, but also weakens their interactions with QCS, thus facilitating more efficient ion transport across the membrane. This relationship between elevated hydration levels and improved diffusion highlights the pivotal role of water in promoting ${\mathrm{OH}}^{-}$ ion mobility within AEMs. At higher hydration levels, water molecules reduce the interaction strength between the ${\mathrm{OH}}^{-}$ ions and the nitrogen atoms of QCS, as demonstrated by the RDF analysis, and form a bicontinuous network, as demonstrated by the simulation snapshots. These observations underscore the critical importance of hydration in optimizing the performance of QCS-based AEMs.

#### 2.2.2. Effect of Temperature

**Figure 7:**
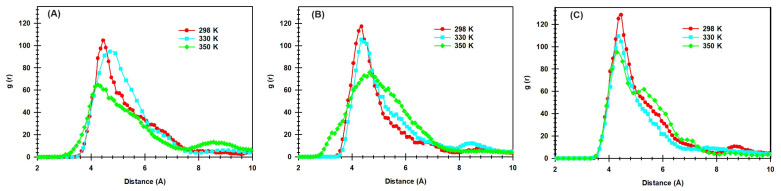
RDFs for the correlation between the oxygen atom of ${\mathrm{OH}}^{-}$ ion and the nitrogen atom of QCS (types **A**–**C**) in the presence of explicit water molecules at the different temperatures.

The effect of temperature on the interaction between ${\mathrm{OH}}^{-}$ ions and QCS head groups was studied using classical all-atom MD simulations at temperatures ranging from 298 K to 350 K. Figure 7 presents the RDF profiles, which quantify the likelihood of finding an oxygen atom of an ion ${\mathrm{OH}}^{-}$ at various distances from nitrogen atoms of different types of QCS (A, B, and C). As the temperature increases, noticeable changes are observed in the RDF peaks. For QCS (A), the peak at 298 K is the highest with a value of 104.7 at 4.4 Å, indicating tighter binding at lower temperatures. As the temperature rises to 330 K and 350 K, the peaks decrease to 94.8 at 4.5 Åand 64.7 at 4.4 Å, respectively, suggesting reduced interaction strength. Similar trends are observed for QCS (B) and QCS (C), with peak values decreasing as temperatures increase, as shown in Figure 7. This trend indicates that higher temperatures lead to a weakening of the interactions between ${\mathrm{OH}}^{-}$ ions and QCS, potentially affecting the dynamics of those species.

**Figure 8:**
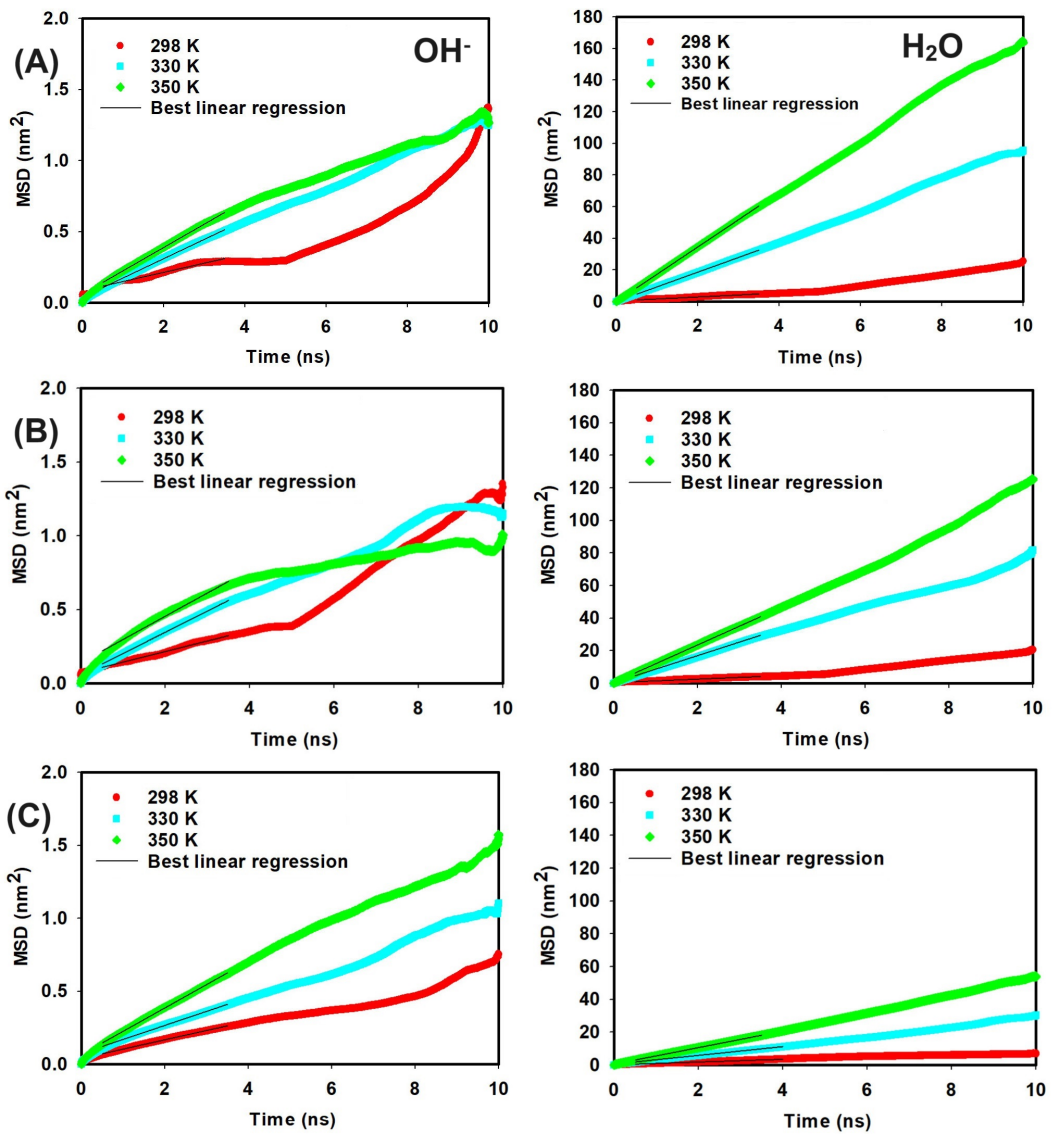
MSD vs. time curves at different temperature values for ${\mathrm{OH}}^{-}$ ions and $H_{2}O$ in systems with QCS types (**A**–**C**), respectively.

To elucidate the impact of temperature on ion mobility, the MSD was analyzed for ${\mathrm{OH}}^{-}$ ions and water molecules at different temperatures. Figure 8 displays the MSD for both components, and the slopes of these curves were used to calculate the diffusion coefficients, presented in Table 5 and Table 6. An increase in temperature from 298 K to 350 K results in higher diffusion coefficients for both ${\mathrm{OH}}^{-}$ ions and water molecules. Specifically, in the presence of QCS (A), the diffusion coefficient of ${\mathrm{OH}}^{-}$ ions increases from 0.011 to 0.027 ${\mathrm{nm}}^{2}/\mathrm{ns}$, and for water molecules from 0.20 to 2.86 ${\mathrm{nm}}^{2}/\mathrm{ns}$. Similar increases are observed for QCS (B) and QCS (C), suggesting that higher temperatures enhance the mobility of both ${\mathrm{OH}}^{-}$ ions and water molecules, as evidenced in Table 5 and Table 6.

This increase in mobility at higher temperatures may be attributed to a change in the dynamics of how molecules and ions interact with each other and with their environment at elevated temperatures. Higher temperatures typically reduce the viscosity of the medium, decrease intermolecular interactions between water molecules, and weaken interactions with QCS functional groups, thereby enhancing ion mobility.

The mobility of ${\mathrm{OH}}^{-}$ ions and water molecules is critical for the efficiency of alkaline fuel cells. Our findings demonstrate that higher temperatures facilitate faster diffusion of these molecules, which is essential for effective ion transport in AEMs. While this study focuses on vehicular transportation of ions, further research incorporating the Grotthuss mechanism, which involves proton hopping facilitated by interactions among water molecules, could provide deeper insights into ion transport dynamics in AEMs. Future investigations will explore these aspects using ab initio molecular dynamics [22,23] to better understand the contributions of different ion transport mechanisms under varying operational conditions.

## 3. Methodology

### 3.1. Computational Models

**Figure 9:**
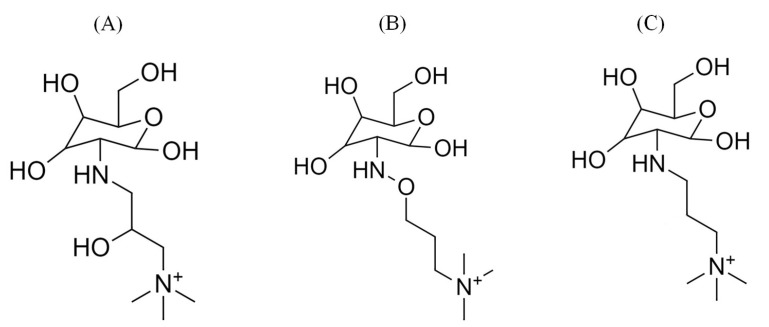
Representative structures of different QCS segments: (**A**) 2-hydroxy propyl trimethyl ammonium chitosan, (**B**) oxy propyl trimethyl ammonium chitosan, and (**C**) propyl trimethyl ammonium chitosan.

Computational models for our DFT-based quantum chemical calculations and classical all-atom MD simulations were constructed based on QCS monomeric structures, designated as A, B, and C, as illustrated in Figure 9. These models draw inspiration from recent experimental works that have explored the synthesis and properties of similar systems [14,24,25,26]. Additional details on the experimental basis for these models can be found in related publications [27,28].

In the DFT calculations, each model comprised a single QCS-based monomeric segment (A, B, or C) associated with one hydroxide ion (${\mathrm{OH}}^{-}$) in the presence of an implicit water environment. This setup was employed to investigate the chemical stability of the monomeric segments and the transport properties of ${\mathrm{OH}}^{-}$ ions within a simulated anion exchange membrane (AEM) environment. Subsequently, for the classical all-atom MD simulations, five monomeric segments of each type (A–C) were combined with five corresponding ${\mathrm{OH}}^{-}$ counterions. The hydration model included a range of 15 to 75 water molecules per ${\mathrm{OH}}^{-}$ ion, reflecting various levels of membrane hydration. The simulations were conducted over a temperature range from 298 K to 350 K, with detailed configurations and initial conditions provided in the Supplementary Materials.

In this study, simulations of monomeric units of QCS were employed to approximate the properties of full polymers, a common approach [12,13,29,30] in computational chemistry where the extensive computational demands of simulating entire polymer chains are a limiting factor. This focused approach targets the QA head groups, where chemical reactions are typically localized and critical to assessing the chemical stability and degradation mechanisms within AEMs. Although this method provides valuable insights into localized chemical behavior, it may not fully capture the broader dynamics of the entire polymer system. Future research will incorporate oligomer simulations to determine how well these localized findings extend to more complex polymer structures.

### 3.2. DFT Calculations

DFT calculations were employed to optimize electronic ground state geometries and to assess molecular electrostatic potential (MEP) maps, bond lengths, LUMO energies distribution, and binding energies ($\Delta E_{\mathrm{binding}}$). Furthermore, these calculations facilitated the identification of transition states and the computation of reaction energies ($\Delta E_{\mathrm{reaction}}$) and activation energies ($\Delta E_{\mathrm{activation}}$). Noncovalent and covalent interactions were analyzed in the intermolecular contacts between the positively charged QCS and the ${\mathrm{OH}}^{-}$ ions in an aqueous phase. We utilized the B3LYP functional combined with the polarizable continuum model (PCM) to optimize the geometries of three QCS segments in both the presence and absence of ${\mathrm{OH}}^{-}$ ions [31,32].

To ascertain MEP, LUMO distribution, LUMO energies, and $\Delta E_{\mathrm{binding}}$, B3LYP 6-311++G(2d,p) level DFT calculations were performed (see Supplementary Materials) [33,34]. Binding energies ($\Delta E_{\mathrm{binding}}$) were determined by the change in total energy when the ${\mathrm{OH}}^{-}$ ions were combined with the QCS segments, as shown in Equation (1):

(1) $$\Delta E_{\mathrm{binding}}=E_{\mathrm{QCS}-\mathrm{OH}}-\left(E_{\mathrm{QCS}}+E_{\mathrm{OH}}\right)$$

For the transition states of the $S_{N}2$ degradation reactions (see Equation (2)) of QCS types A, B, and C in the presence of ${\mathrm{OH}}^{-}$ ions and explicit water molecules at a hydration level of 3, optimizations were performed using B3LYP 6-311++G(2d,p) in implicit DMSO.

(2) $$R-N^{+}{\left({\mathrm{CH}}_{3}\right)}_{3}+{\mathrm{OH}}^{-}{\left(H_{2}O\right)}_{3}\to R-\mathrm{OH}+N{\left({\mathrm{CH}}_{3}\right)}_{3}+3H_{2}O$$

where the R group represents $C_{9}H_{16}O_{6}$ for QCS (A) and QCS (B), and $C_{9}H_{16}O_{5}$ for QCS (C).

**Figure 10:**
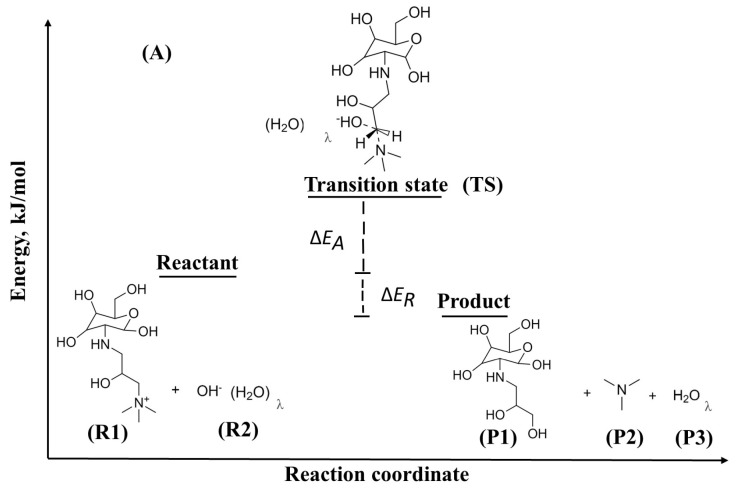
Illustration of the representative segment of QCS (A) for the $S_{N}2$ degradation reaction.

As illustrated in Figure 10, $\Delta E_{\mathrm{reaction}}$ and $\Delta E_{\mathrm{activation}}$ for these reactions are given by

(3) $$\Delta E_{\mathrm{reaction}}=\sum E_{\mathrm{products}}-\sum E_{\mathrm{reactants}}$$

(4) $$\Delta E_{\mathrm{activation}}=E_{\mathrm{transition}\mathrm{state}}-(\sum E_{\mathrm{reactants}}+E_{\mathrm{BSSE}})$$

For these calculations, all systems were modeled in the implicit DMSO solvent with a dielectric constant of 46.83. The calculations were further refined using a water cluster model in our PCM to enhance accuracy by minimizing discrepancies in solvation effects.

The low values of $\Delta E_{\mathrm{activation}}$ suggest that the QCS segments are more susceptible to degradation by ${\mathrm{OH}}^{-}$ ions. The correctness of all stationary points as absolute minima on their respective potential energy surfaces was confirmed by further calculations of the second derivatives of the energy. All DFT analyses and calculations were conducted using the GaussView (v6.0) software and GAUSSIAN16 suite [35].

### 3.3. Classical All-Atom MD Simulation

Classical all-atom MD simulations were performed to investigate the behavior of a monomeric unit of QCS and hydroxide ion (${\mathrm{OH}}^{-}$) in water, representative of the environments of the anion exchange membrane (AEM) as shown in Figure 9. Initial topology and force field parameters for the QCS segments were derived using SwissParam, configured for compatibility with the CHARMM36 force field [36,37]. For the simulation, QCS head groups were modeled using standard CHARMM36 parameters, while water molecules were represented using the TIP3P water model [38]. Parameters for the hydroxide ion were adapted from Han et al. [39], applying approaches consistent with those used in similar polymer systems by Zhang et al. [18].

The initial setup of the simulated systems consists of QCS, ${\mathrm{OH}}^{-}$ ions, and water molecules adjusted to hydration levels (HL) of 3, 9, and 15 and temperatures ranging from 298 to 350 K, detailed in the Supplementary Materials. The system’s charge neutrality was maintained by adjusting the number of ${\mathrm{OH}}^{-}$ ions, with the number of water molecules adjusted to the specified HL.

The simulation protocol began with an energy minimization using the steepest descent method, limiting the maximum force to 500 kJ/mol/nm, followed by 1 ns of NPT equilibration at each temperature setting (298, 330, and 350 K) and 1 bar pressure. Subsequent NVT equilibration at the same temperatures ensured system stability before running production MD simulations for 10 ns under the NVT ensemble.

The LINCS algorithm was employed to constrain all bonds. Short-range interactions were treated with a 1.0 nm cut-off for Lennard-Jones and Coulombic interactions, while long-range electrostatics were handled via the particle mesh Ewald summation method, utilizing a 0.14 nm grid spacing and fourth-order interpolation [40]. Temperature control was achieved with the Nose–Hoover thermostat [41] with a coupling constant of 0.2 ps. Pressure control was achieved using the Berendsen barostat [42] with isotropic pressure coupling and a coupling constant of 0.5 ps, and a reference pressure of 1 bar. Periodic boundary conditions were applied in all directions. These settings align with commonly used parameters in the field.

The simulation densities were calculated as 1.14 g/${\mathrm{cm}}^{3}$, 1.09 g/${\mathrm{cm}}^{3}$, and 1.07 g/${\mathrm{cm}}^{3}$ for HLs of 3, 9, and 15, respectively, aligning closely with the experimental values reported in the literature [18].

The simulations were executed using GROMACS 2021.4 [43], with system visualization and analysis of radial distribution functions (RDFs), mean square displacement (MSD), and diffusion coefficients performed using visualization molecular dynamics (VMD) software [44].

## 4. Conclusions

This study conducted a computational analysis of the chemical stability and ion transport properties of several functional groups in QCS-based anion exchange membranes using a combination of DFT-based quantum chemical calculations and all-atom MD simulations. The DFT calculations were instrumental in elucidating the electronic structure, revealing an increasing trend in chemical stability among the QCS variants: propyl trimethyl ammonium chitosan (C) < oxy propyl trimethyl ammonium chitosan (B) < 2-hydroxy propyl trimethyl ammonium chitosan (A) at hydration levels (HL) of 0 and 3. This trend is supported by the calculated LUMO energies, reaction energies, and activation energies, which collectively highlight the superior stability of the 2-hydroxy propyl trimethyl ammonium chitosan (A) variant under varying hydration conditions.

Further exploration through all-atom MD simulations provided a dynamic view of the ion transport mechanisms across different hydration levels and temperatures. The diffusion coefficients of ${\mathrm{OH}}^{-}$ ions for all three QCS variants were observed to increase with hydration level and temperature, suggesting that higher hydration levels and temperatures enhance ion mobility across the membrane. The comparable diffusion coefficients across the three variants underscore their effectiveness in the vehicular transport of ${\mathrm{OH}}^{-}$ ions in AEM applications.

Comparing our results with other theoretical studies and experimental data in the literature, our previous work [17,45] utilizing DFT and classical MD methods highlighted the chemical stability and ${\mathrm{OH}}^{-}$ ion diffusion characteristics of various QA head groups. Compared to those head groups explored in our previous work, QCS-A demonstrated higher stability, in agreement with recent experimental studies [46,47] which also identified QCS-A as exhibiting higher LUMO energies and activation energies, indicating a greater resistance to ${\mathrm{OH}}^{-}$ ion attack. Furthermore, the diffusion coefficients of the ${\mathrm{OH}}^{-}$ ions obtained from our MD simulations are consistent with values reported in the literature [18,19], thus validating our computational methods. Although direct experimental comparisons between QCS-A and its variants (B and C) remain unavailable, the consistency of our theoretical findings with established experimental data on similar systems supports the reliability of our results.

These findings not only confirm the crucial role of structural variations between QCS variants in determining the stability and performance of AEMs but also underscore the importance of operational conditions such as hydration level and temperature in optimizing membrane performance. The study contributes valuable insights into the design and development of AEMs, offering guidance for the selection of QCS materials on the basis of the desired balance of stability and ion transport efficiency. Future work will focus on extending these analyses to include the impact of other environmental factors, with the aim of developing more robust and efficient AEMs for sustainable energy applications.

## Figures and Tables

**Table 1:** Reaction and activation energy values for our designed systems, measured at 298 K and 1 bar at the HL 0. Units: kJ/mol.

QCS	$\Delta E_{\mathrm{reaction}}$	BSSE	$\Delta E_{\mathrm{activation}}$
(A)	−123.48	15.12	66.51
(B)	−130.62	16.11	37.01
(C)	−132.94	14.72	36.21

**Table 2:** Reaction and activation energy values for our designed systems, measured at 298 K and 1 bar at the HL 3. Units: kJ/mol.

QCS	$\Delta E_{\mathrm{reaction}}$	BSSE	$\Delta E_{\mathrm{activation}}$
(A)	−21.56	11.47	147.27
(B)	−28.69	8.38	103.20
(C)	−31.02	8.47	89.92

**Table 3:** Diffusion coefficients of ${\mathrm{OH}}^{-}$ ions across different QCS structures and HLs.

*D*, ${\mathrm{nm}}^{2}/\mathrm{ns}$, (SE)		HL Values		
		3	9	15
${\mathrm{OH}}^{-}$	(A)	0.011 (0.004)	0.017 (0.005)	0.027 (0.003)
	(B)	0.011 (0.003)	0.016 (0.006)	0.026 (0.002)
	(C)	0.011 (0.001)	0.015 (0.002)	0.026 (0.002)

**Table 4:** Diffusion coefficients of $H_{2}O$ molecules across different QCS structures and HLs.

*D*, ${\mathrm{nm}}^{2}/\mathrm{ns}$, (SE)		HL Values		
		3	9	15
$H_{2}O$	(A)	0.20 (0.07)	0.40 (0.06)	0.46 (0.04)
	(B)	0.18 (0.04)	0.39 (0.05)	0.43 (0.06)
	(C)	0.13 (0.03)	0.39 (0.02)	0.42 (0.05)

**Table 5:** Diffusion coefficients of ${\mathrm{OH}}^{-}$ ions across various QCS structures at different temperatures.

*D*, ${\mathrm{nm}}^{2}/\mathrm{ns}$, (SE)		Temperature Values (K)		
		298	330	350
${\mathrm{OH}}^{-}$	(A)	0.011 (0.004)	0.023 (0.003)	0.027 (0.003)
	(B)	0.011 (0.003)	0.023 (0.003)	0.026 (0.003)
	(C)	0.011 (0.001)	0.016 (0.002)	0.026 (0.003)

**Table 6:** Diffusion coefficients of $H_{2}O$ molecules across various QCS structures at different temperatures.

*D*, ${\mathrm{nm}}^{2}/\mathrm{ns}$ (SE)		Temperature Values (K)		
		298	330	350
$H_{2}O$	(A)	0.20 (0.07)	1.54 (0.04)	2.86 (0.25)
	(B)	0.18 (0.04)	1.38 (0.06)	1.92 (0.20)
	(C)	0.13 (0.03)	0.44 (0.05)	0.84 (0.09)

## Data Availability

The data and materials are available from the authors upon request.

## Supplementary Materials

The following supporting information can be downloaded at: https://www.mdpi.com/article/10.3390/molecules29133175/s1, The Supplementary Material includes extended DFT results, MD settings, and detailed MSD vs. time curves for ${\mathrm{OH}}^{-}$ ions and water across different QCS types and conditions; Figure S1: Structure of representative segments of QCS (A) were illustrated for the $S_{N}2$ degradation reaction; Figure S2: Structure of representative segments of QCS (B) were illustrated for the $S_{N}2$ degradation reaction; Figure S3: Structure of representative segments of QCS (C) were illustrated for the $S_{N}2$ degradation reaction; Figure S4: Representation of molecular electrostatic potential maps for various QCS segments, and their complexes with ${\mathrm{OH}}^{-}$ ions; Figure S5: Complexes of three different QCS segments with ${\mathrm{OH}}^{-}$ ions. Color key: white (hydrogen); grey (carbon); blue (nitrogen); green (chloride); Figure S6: MSD plot and the trend line at the HL 3 for ${\mathrm{OH}}^{-}$ ions in the presence of QCS (A) of AEM; Figure S7: MSD plot and the trend line at the HL 9 for ${\mathrm{OH}}^{-}$ ions in the presence of QCS (A) of AEM; Figure S8: MSD plot and the trend line at the HL 15 for ${\mathrm{OH}}^{-}$ ions in the presence of QCS (A) of AEM; Figure S9: MSD plot and the trend line at the HL 3 for ${\mathrm{OH}}^{-}$ ions in the presence of QCS (B) of AEM; Figure S10: MSD plot and the trend line at the HL 9 for ${\mathrm{OH}}^{-}$ ions in the presence of QCS (B) of AEM; Figure S11: MSD plot and the trend line at the HL 15 for ${\mathrm{OH}}^{-}$ ions in the presence of QCS (B) of AEM.; Figure S12: MSD plot and the trend line at the HL 3 for ${\mathrm{OH}}^{-}$ ions in the presence of QCS (C) of AEM; Figure S13: MSD plot and the trend line at the HL 9 for ${\mathrm{OH}}^{-}$ ions in the presence of QCS (C) of AEM; Figure S14: MSD plot and the trend line at the HL 15 for ${\mathrm{OH}}^{-}$ ions in the presence of QCS (C) of AEM; Figure S15: MSD plot and the trend line at the HL 3 for $H_{2}O$ ions in the presence of QCS (A) of AEM; Figure S16: MSD plot and the trend line at the HL 9 for $H_{2}O$ ions in the presence of QCS (A) of AEM; Figure S17: MSD plot and the trend line at the HL 15 for $H_{2}O$ ions in the presence of QCS (A) of AEM; Figure S18: MSD plot and the trend line at the HL 3 for $H_{2}O$ ions in the presence of QCS (B) of AEM; Figure S19: MSD plot and the trend line at the HL 9 for $H_{2}O$ ions in the presence of QCS (B) of AEM; Figure S20: MSD plot and the trend line at the HL 15 for $H_{2}O$ ions in the presence of QCS (B) of AEM; Figure S21: MSD plot and the trend line at the HL 3 for $H_{2}O$ ions in the presence of QCS (C) of AEM; Figure S22: MSD plot and the trend line at the HL 9 for $H_{2}O$ ions in the presence of QCS (C) of AEM; Figure S23: MSD plot and the trend line at the HL 15 for $H_{2}O$ ions in the presence of QCS (C) of AEM; Figure S24: MSD plot and the trend line at the HL 3 for ${\mathrm{OH}}^{-}$ ions in the presence of QCS (A) of AEM at 298 K; Figure S25: MSD plot and the trend line at the HL 3 for ${\mathrm{OH}}^{-}$ ions in the presence of QCS (A) of AEM at the 330 K; Figure S26: MSD plot and the trend line at the HL 3 for ${\mathrm{OH}}^{-}$ ions in the presence of QCS (A) of AEM at the 350 K; Figure S27: MSD plot and the trend line at the HL 3 for ${\mathrm{OH}}^{-}$ ions in the presence of QCS (B) of AEM at the 298 K; Figure S28: MSD plot and the trend line at the HL 3 for ${\mathrm{OH}}^{-}$ ions in the presence of QCS (B) of AEM at the 330 K; Figure S29: MSD plot and the trend line at the HL 3 for ${\mathrm{OH}}^{-}$ ions in the presence of QCS (B) of AEM at the 350 K; Figure S30: MSD plot and the trend line at the HL 3 for ${\mathrm{OH}}^{-}$ ions in the presence of QCS (C) of AEM at the 298 K; Figure S31: MSD plot and the trend line at the HL 3 for ${\mathrm{OH}}^{-}$ ions in the presence of QCS (C) of AEM at the 330 K; Figure S32: MSD plot and the trend line at the HL 3 for ${\mathrm{OH}}^{-}$ ions in the presence of QCS (C) of AEM at the 350 K; Figure S33: MSD plot and the trend line at the HL 3 for $H_{2}O$ ions in the presence of QCS (A) of AEM at the 298 K; Figure S34: MSD plot and the trend line at the HL 3 for $H_{2}O$ ions in the presence of QCS (A) of AEM at the 330 K; Figure S35: MSD plot and the trend line at the HL 3 for $H_{2}O$ ions in the presence of QCS (A) of AEM at the 350 K; Figure S36: MSD plot and the trend line at the HL 3 for $H_{2}O$ ions in the presence of QCS (B) of AEM at the 298 K; Figure S37: MSD plot and the trend line at the HL 3 for $H_{2}O$ ions in the presence of QCS (B) of AEM at the 330 K; Figure S38: MSD plot and the trend line at the HL 3 for $H_{2}O$ ions in the presence of QCS (B) of AEM at the 350 K; Figure S39: MSD plot and the trend line at the HL 3 for $H_{2}O$ ions in the presence of QCS (C) of AEM at the 298 K; Figure S40: MSD plot and the trend line at the HL 3 for $H_{2}O$ ions in the presence of QCS (C) of AEM at the 330 K; Figure S41: MSD plot and the trend line at the HL 3 for $H_{2}O$ ions in the presence of QCS (C) of AEM at 350 K; Table S1: Description for our designed systems at 298 K and 1 bar; Table S2: Energy values for our designed systems at 298 K and 1 bar; Table S3: Energy values for our designed systems of AEM studied by the DFT calculations at the HL 0; Table S4: Energy values for our designed systems of AEM studied by the DFT calculations at the HL 3.

## Abbreviations

The following abbreviations are used in this manuscript:

AEM	Anion Exchange Membrane
AEMFCs	Anion Exchange Membrane Fuel Cells
AFCs	Alkaline Fuel Cells
BSSE	Basis set superposition error
DFT	Density Functional Theory
$\Delta E_{\mathrm{reaction}}$	Reaction energy
$\Delta E_{\mathrm{activation}}$	Activation energy
$\Delta E_{\mathrm{binding}}$	Binding energy
DMFCs	Direct Methanol Fuel Cells
EFCs	Enzymatic Fuel Cells
HL	Hydration level
${\mathrm{OH}}^{-}$	Hydroxide ion
LUMO	Lowest unoccupied molecular orbital
MCFCs	Molten Carbonate Fuel Cells
MD	Molecular dynamics
MSD	Mean square displacement
$S_{N}2$	Nucleophilic substitution
PAFCs	Phosphoric Acid Fuel Cells
PCM	Polarizable continuum model
PEMFCs	Proton Exchange Membrane Fuel Cells
QCS	Quaternized chitosan
QA	Quaternary ammonium
RDF	Radial distribution function
SOFCs	Solid Oxide Fuel Cells
