# Supplementary material for: Atomistic Modeling of Quaternized Chitosan Head Groups: Insights into Chemical Stability and Ion Transport for Anion Exchange Membrane Applications
Source: Molecules. 2024 Jul 3;29(13):3175. doi: 10.3390/molecules29133175 (PMC11243541; doi:10.3390/molecules29133175)
Supplement: Supplementary file 1 [file molecules-29-03175-s001.zip › molecules-3031636-supplementary.pdf]

Supporting Materials for "Atomistic Modeling of Quaternized Chitosan Head Groups: Insights into Chemical Stability and Ion Transport for Anion Exchange Membrane Applications"

Mirat Karibayev[a], Bauyrzhan Myrzakhmetov[b], Dias Bekeshov[a], Yanwei Wang[a,b,*], Almagul Mentbayeva[a,*]

[a]Department of Chemical and Materials Engineering, School of Engineering and Digital Sciences, Nazarbayev University, 53 Kabanbay Batyr Avenue, Astana, 010000, Kazakhstan
[b]Center for Energy and Advanced Materials Science, National Laboratory Astana, 53 Kabanbay Batyr Avenue, Astana, 010000, Kazakhstan
[*]Correspondence: yanwei.wang@nu.edu.kz (Y.W.); almagul.mentbayeva@nu.edu.kz (A.M.)

This Supplementary Material provides detailed supplementary data to support the findings of this study, including additional simulation settings (section S1), extended Density Functional Theory results (section S2), and supplemental Molecular Dynamics findings (section S3). Key elements include configurations and parameters used for simulations, molecular electrostatic potential maps, and radial distribution functions. Detailed Mean Square Displacement (MSD) vs. time curves from repeated simulation runs are also provided, illustrating the diffusivity of hydroxide ions and water molecules at various hydration levels and temperatures across different QCS configurations.

S1. Additional simulation settings

The transition states for the S_N2 degradation reactions of the QCS (A), QCS (B), and QCS (C) in the presence of OH^- ions and explicit water molecules at the hydration level (HL) 3 were presented in Figures S1 to S3.

The standard all-atom MD simulation setup was created by placing five QCS (labeled A-C) and five negatively charged OH^- ions, along with 15, 45, or 75 water molecules at 298 K, as shown in Table S1.

A theoretical model of QCS (A), (B), and (C) of AEM was established by using 5 QCS, 5 OH⁻ ions, and 15 water molecules to study the impact of the temperatures from 298 K to 350 K.

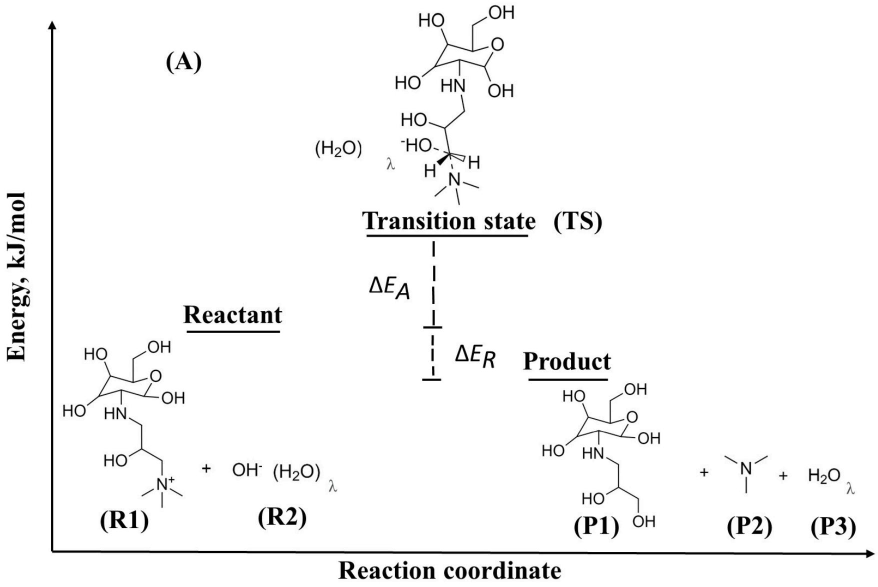

Figure S1: Structure of representative segments of QCS (A) were illustrated for the S_N2 degradation reaction.

S2. Supplementary Results from DFT calculations

Figure S4 shows the MEP maps for the binding of QCS segments with OH^- ions and the charge distribution. The positive charge of the QCS segment was stabilized by placing OH^- ion near the nitrogen atom. The net charges of OH^- ions and QCS segments are -1 and +1, respectively. It is evident that OH^- ions stabilize the positive charge of QCS of the AEM by networking with the nitrogen atom.

OH^- ions produced by the oxygen reduction process on the cathode catalyst's surface are transported to the anode through the AEM. Figure S5 shows the structures where the OH^- ions arrive at the electrolyte membrane (AEM) in implicit water solvation. Through three branched H-bonds between the OH^- ion and the QCS of AEM, the OH^- ion was positioned close to the distinct QCS of AEM in the structures. As a result, calculation of

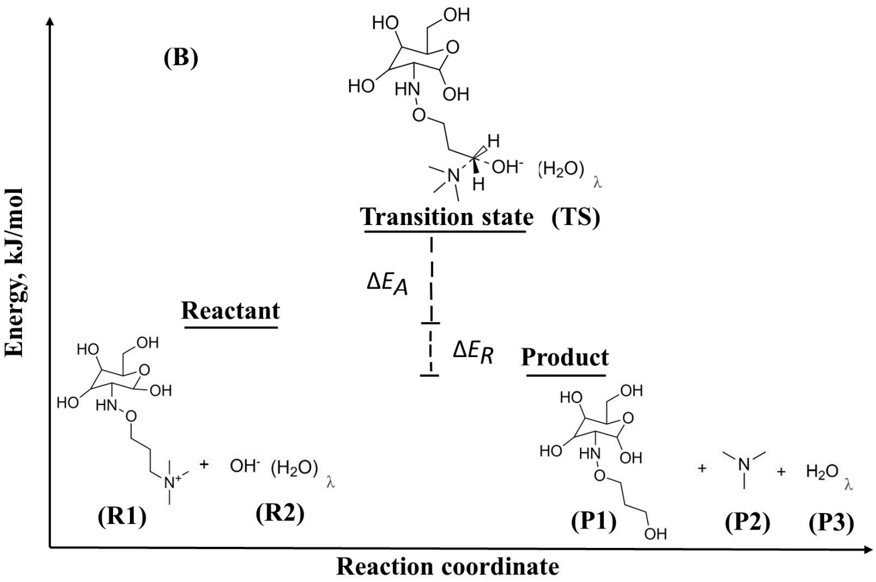

Figure S2: Structure of representative segments of QCS (B) were illustrated for the S_N2 degradation reaction.

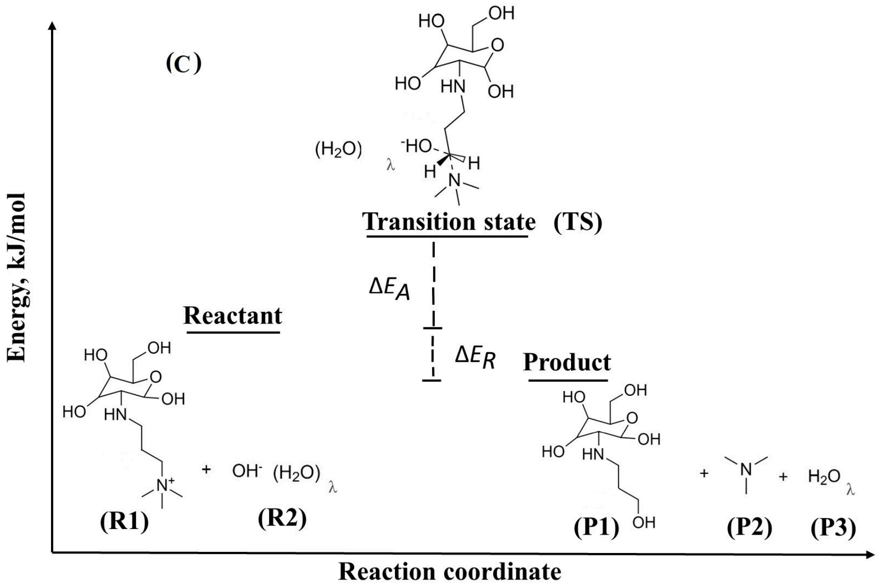

Figure S3: Structure of representative segments of QCS (C) were illustrated for the S_N2 degradation reaction.

Table S1: Description for our designed systems at 298 K and 1 bar.

	AEM	OH^-	Water	Number of atoms	Box size
(A)	5	5	15	290	$1.45 \times 1.45 \times 1.45$ nm^3
(A)	5	5	45	380	$1.61 \times 1.61 \times 1.61$ nm^3
(A)	5	5	75	470	$1.74 \times 1.74 \times 1.74$ nm^3
(B)	5	5	15	290	$1.45 \times 1.45 \times 1.45$ nm^3
(B)	5	5	45	380	$1.61 \times 1.61 \times 1.61$ nm^3
(B)	5	5	75	470	$1.74 \times 1.74 \times 1.74$ nm^3
(C)	5	5	15	285	$1.44 \times 1.44 \times 1.44$ nm^3
(C)	5	5	55	375	$1.60 \times 1.60 \times 1.60$ nm^3
(C)	5	5	75	465	$1.73 \times 1.73 \times 1.73$ nm^3

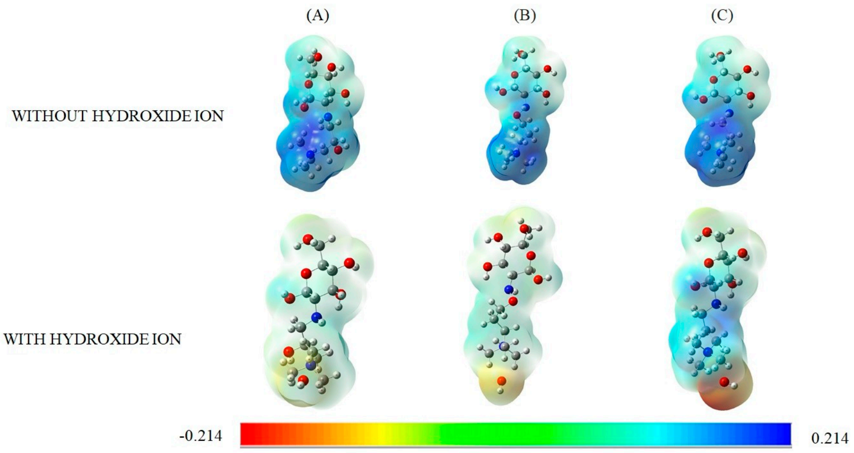

Figure S4: Representation of molecular electrostatic potential maps for various QCS seg- ments, and their complexes with OH^- ions.

$\Delta E_{binding}$ by B3LYP DFT was implemented to characterize the OH^- ion transportation process in AEMs (see Table S2 and Figure S5.)

$\Delta E_{binding}$ of several QCS of AEM segments was calculated from the optimized structures by adding implicit water, and OH^- ions were introduced explicitly. As previously mentioned, the positive charge was stabilized by

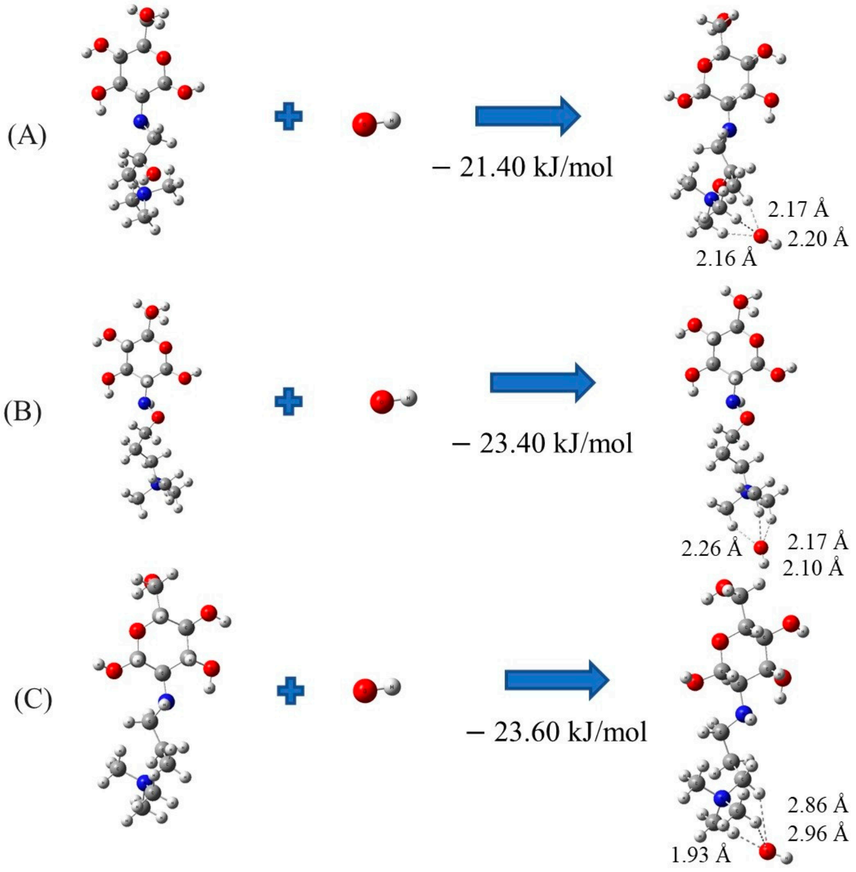

Figure S5: Complexes of three different QCS segments with OH^- ions. Color key: white (hydrogen); grey (carbon); blue (nitrogen); green (chloride).

Table S2: Energy values for our designed systems at 298 K and 1 bar.

QCS	E(OH) (kJ/mol)	E(QA) (kJ/mol)	E(QA with OH) (kJ/mol)	$\Delta E_{binding}$
(A)	-199432.19	-2716167.23	-2915620.89	-21.40
(B)	-199432.19	-2716040.05	-2915495.66	-23.40
(C)	-199432.19	-2518620.95	-2718076.76	-23.60

locating OH^- ions near the nitrogen atoms of the QCS of AEM segments. Table S2 and Figure S5 display the outcomes of the corresponding $\Delta E_{binding}$ with optimal structures for complexes of OH^- ions with the different QCS of AEM. The order of the binding strength of OH^- ions with the different QCS of AEM may be seen from the $\Delta E_{binding}$ data as follows: (A) > (B) ∼ (C). Herein, the higher value of $\Delta E_{binding}$ indicates the strong interaction, which could transfer OH^- ions to the positively charged nitrogen atoms automatically promoted by the formation of three hydrogen bonds. However, to cross the $\Delta E_{binding}$ values, OH^- ions must travel rapidly from one side of the QA head group of AEM to the other. In this regard, increased binding strength equates to decreased OH^- ion transport via the QA head groups of AEM, leading to lower conductivity. Additionally, the DFT study of binding energy suggested that the 2-hydroxy propyl trimethyl ammonium chitosan structure might improve the ionic conductivity (IC) among various quaternized chitosan structures.

The values of reactant, product, and transition state energies for our designed systems of AEM studied by the DFT calculations are listed in Tables S3 and S4.

Table S3: Energy values for our designed systems of AEM studied by the DFT calculations at the HL 0.

	E(R1)	E(R2)	E(TS)	E(P1)	E(P2)
(A)	-2716186.58	-199432.19	-2915537.13	-2457504.02	-458238.23
(B)	-2716037.74	-199432.19	-2915417.54	-2457362.32	-458238.23
(C)	-2518618.59	-199432.19	-2717999.84	-2259945.49	-458238.23

Table S4: Energy values for our designed systems of AEM studied by the DFT calculations at the HL 3.

	E(R1)	E(R2)	E(TS)	E(P1)	E(P2)	E(P3)
(A)	-2716186.58	-801864.08	-3517891.91	-2457504.02	-458238.23	-602329.96
(B)	-2716037.74	-801864.08	-3517790.24	-2457362.32	-458238.23	-602329.96
(C)	-2518618.59	-801864.08	-3320373.26	-2259945.49	-458238.23	-602329.96

S3. Supplementary Results from MD simulations

S3.1 MSD vs. time curves from repeated runs

In our study, the calculation of hydroxide ion and water diffusivity involved the analysis of the mean square displacement (MSD) and was determined using Einstein's relation:

$$D = \frac{1}{6t} R^2 \quad \text{(s1)}$$

To estimate the diffusion coefficient, a 10 ns simulation run was conducted. To minimize statistical uncertainty, we carried out 10 independent simulations for each system design. The diffusion coefficients for hydroxide ions and water molecules were derived from the slope of the MSD versus time curve. The MSD for OH^- ions and H_2O at various QCS structures, at the different HLs, and at the different temperatures are depicted in Figure S6 to S41.

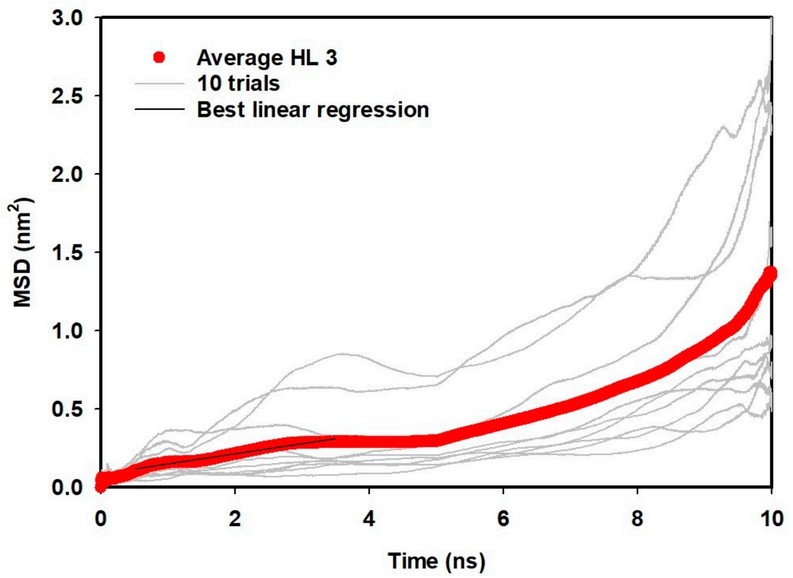

Figure S6: MSD plot and the trend line at the HL 3 for OH^- ions in the presence of QCS (A) of AEM.

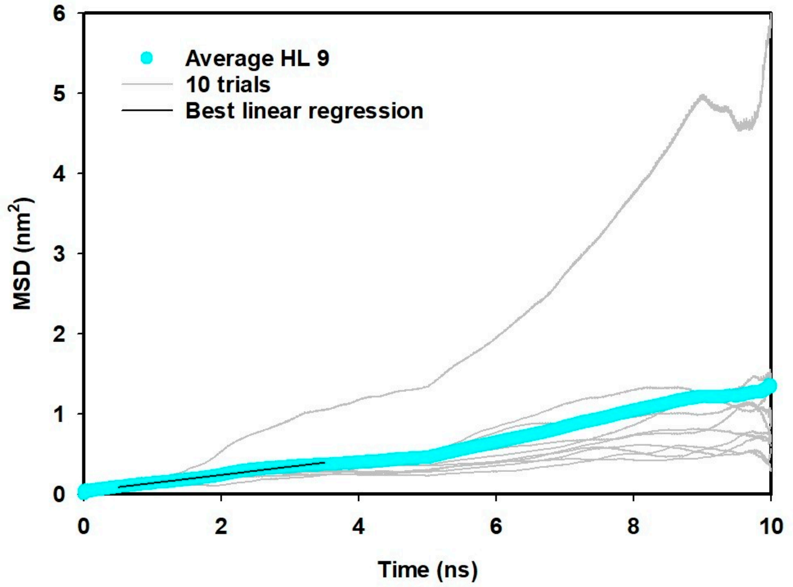

Figure S7: MSD plot and the trend line at the HL 9 for OH^- ions in the presence of QCS (A) of AEM.

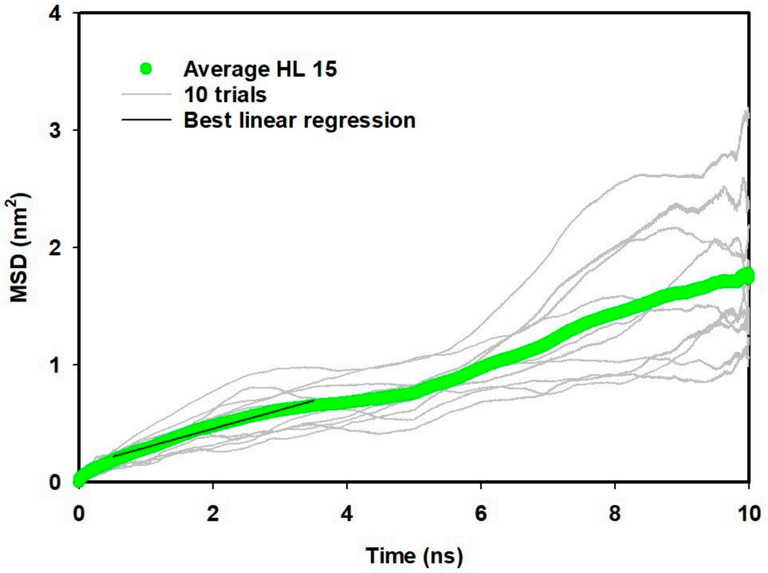

Figure S8: MSD plot and the trend line at the HL 15 for OH^- ions in the presence of QCS (A) of AEM.

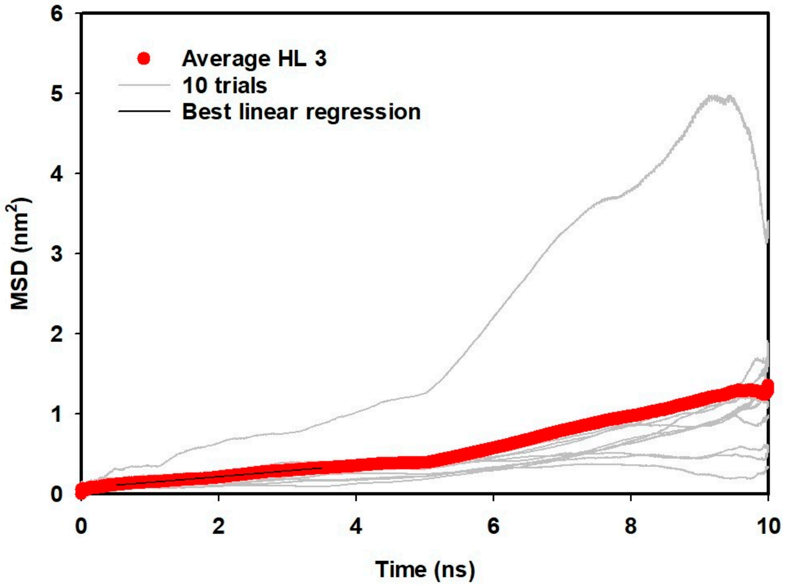

Figure S9: MSD plot and the trend line at the HL 3 for OH^- ions in the presence of QCS (B) of AEM.

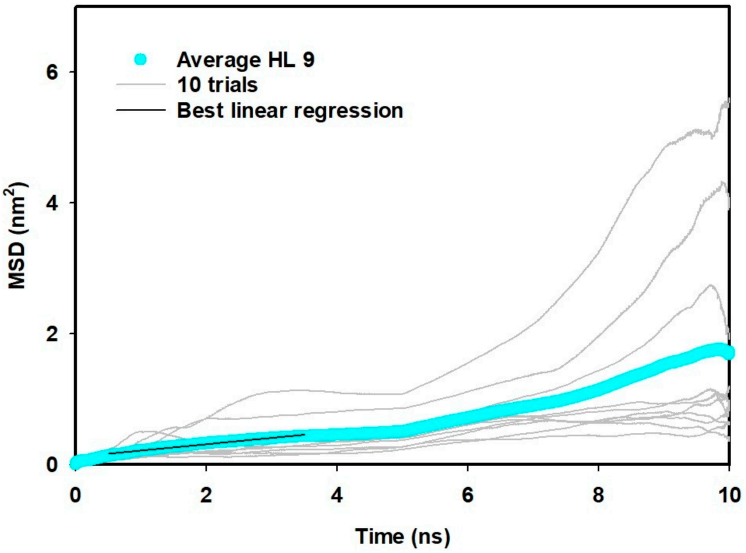

Figure S10: MSD plot and the trend line at the HL 9 for OH^- ions in the presence of QCS (B) of AEM.

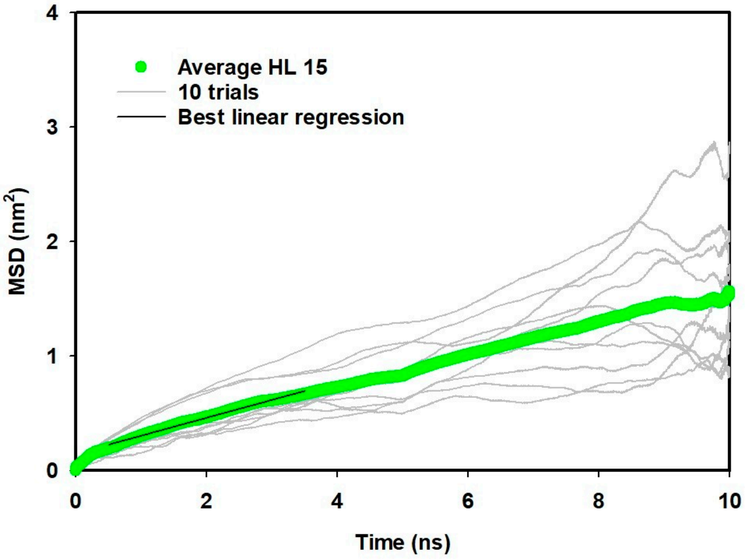

Figure S11: MSD plot and the trend line at the HL 15 for OH^- ions in the presence of QCS (B) of AEM.

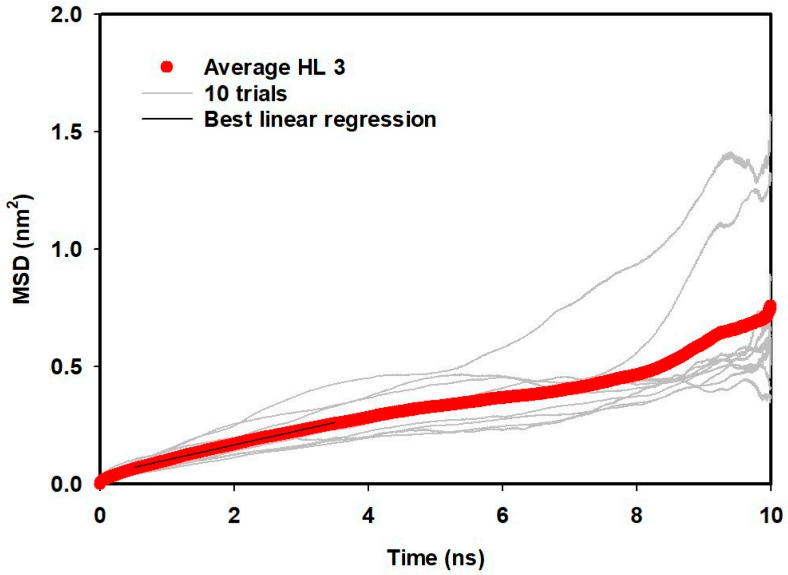

Figure S12: MSD plot and the trend line at the HL 3 for OH^- ions in the presence of QCS (C) of AEM.

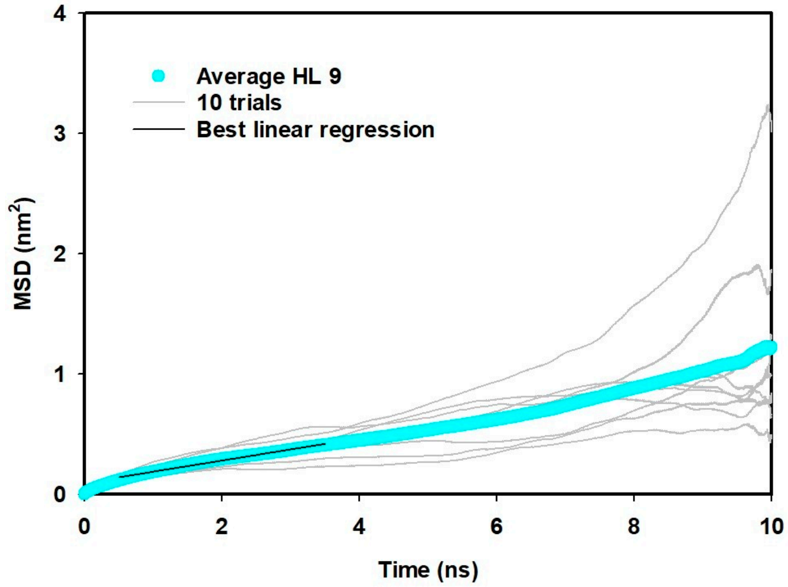

Figure S13: MSD plot and the trend line at the HL 9 for OH^- ions in the presence of QCS (C) of AEM.

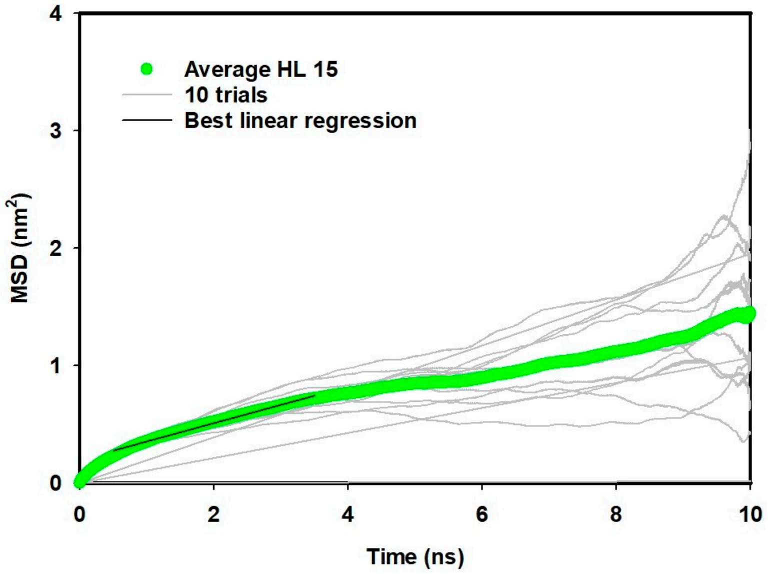

Figure S14: MSD plot and the trend line at the HL 15 for OH^- ions in the presence of QCS (C) of AEM.

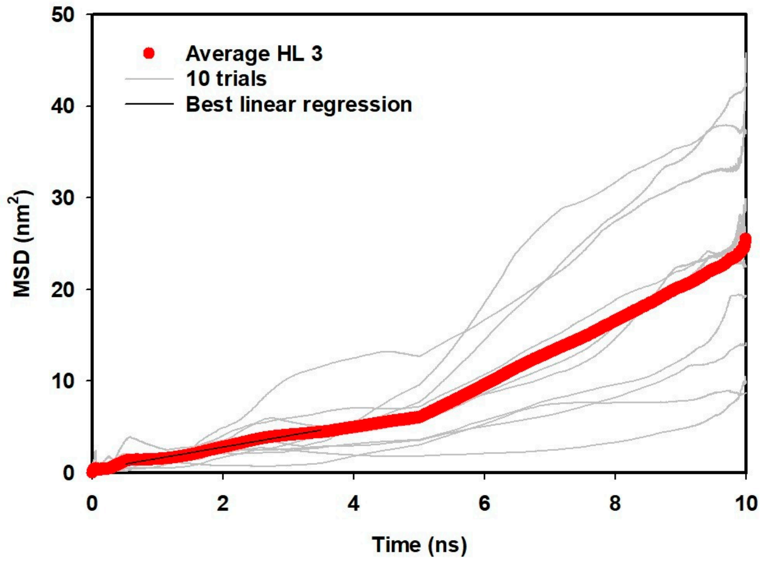

Figure S15: MSD plot and the trend line at the HL 3 for H_2O ions in the presence of QCS (A) of AEM.

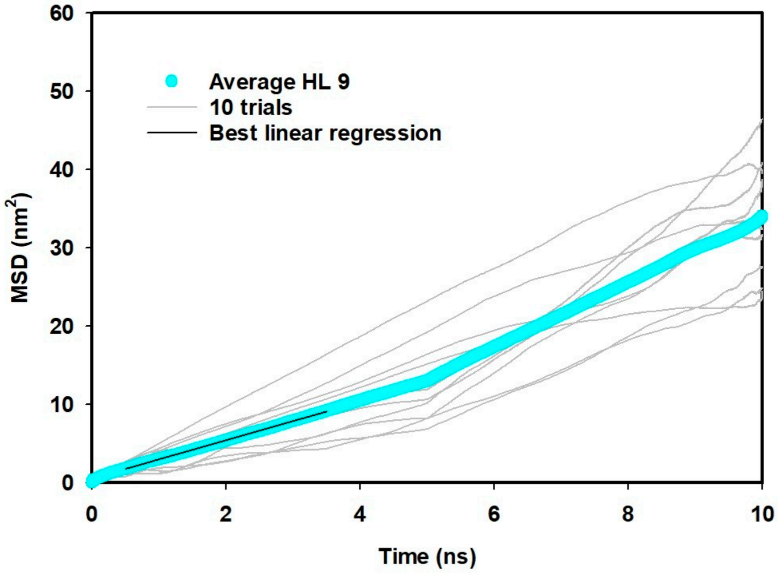

Figure S16: MSD plot and the trend line at the HL 9 for H_2O ions in the presence of QCS (A) of AEM.

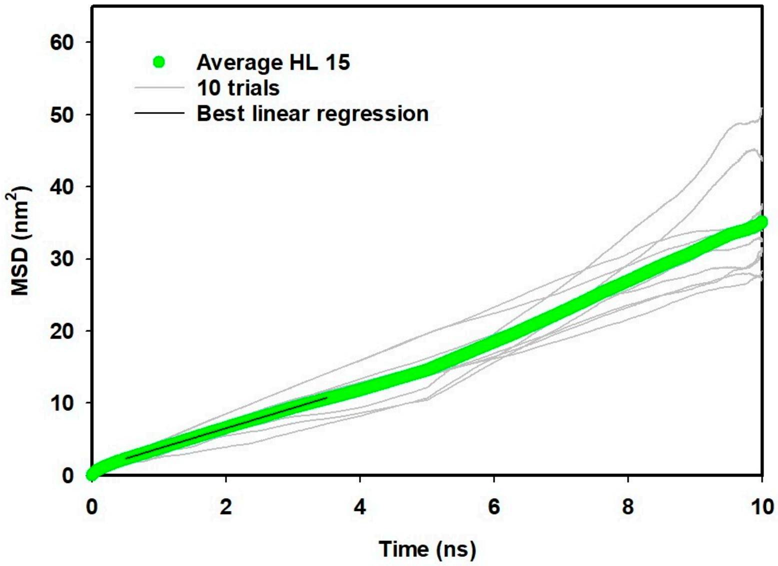

Figure S17: MSD plot and the trend line at the HL 15 for H_2O ions in the presence of QCS (A) of AEM.

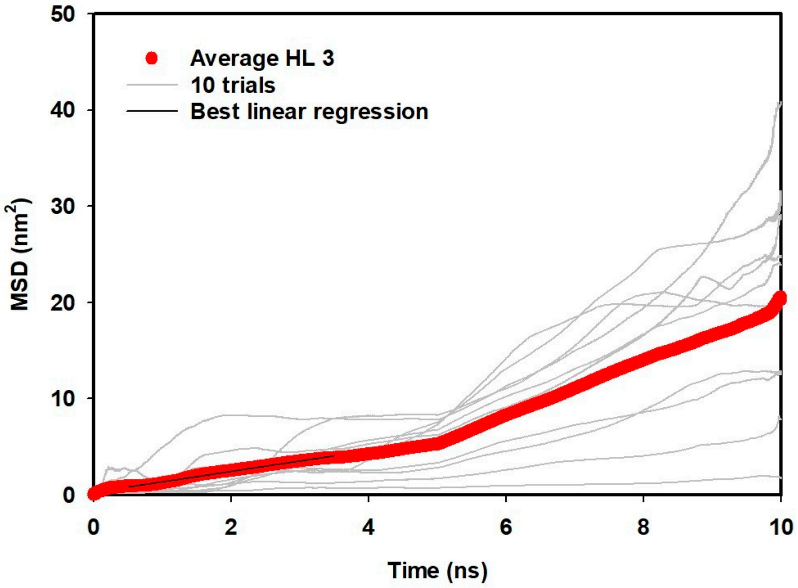

Figure S18: MSD plot and the trend line at the HL 3 for H_2O ions in the presence of QCS (B) of AEM.

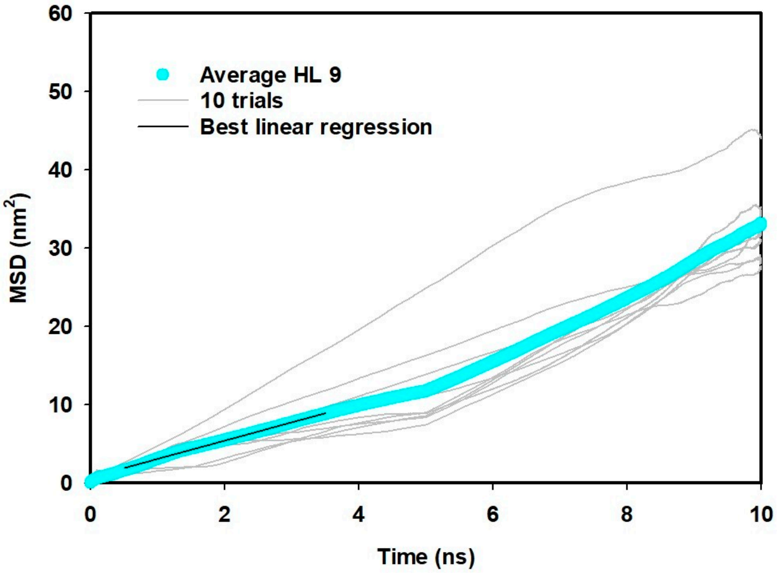

Figure S19: MSD plot and the trend line at the HL 9 for H_2O ions in the presence of QCS (B) of AEM.

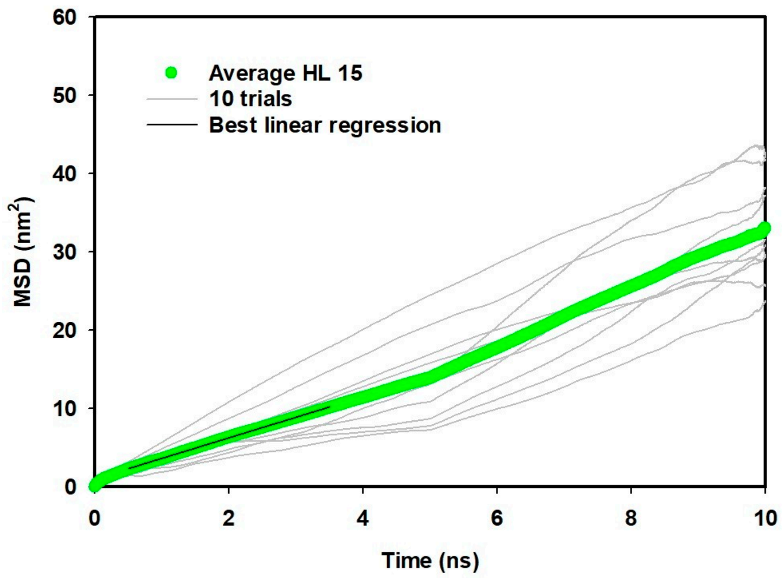

Figure S20: MSD plot and the trend line at the HL 15 for H_2O ions in the presence of QCS (B) of AEM.

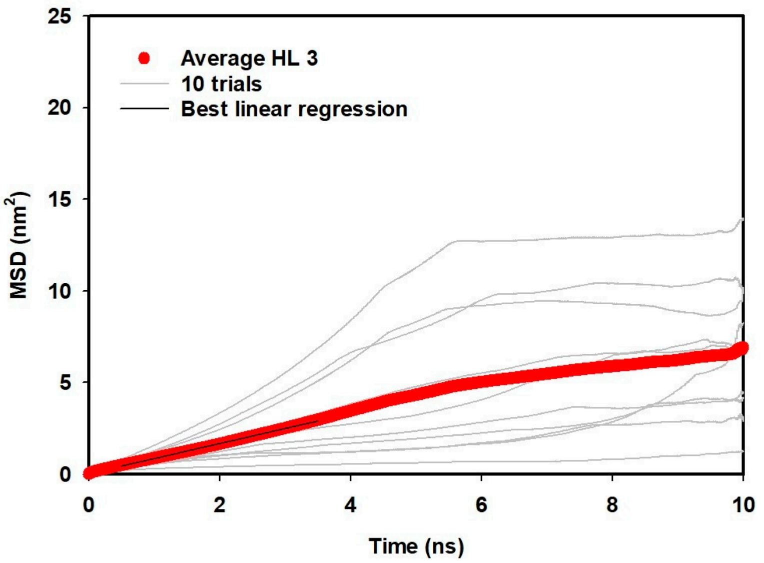

Figure S21: MSD plot and the trend line at the HL 3 for H_2O ions in the presence of QCS (C) of AEM.

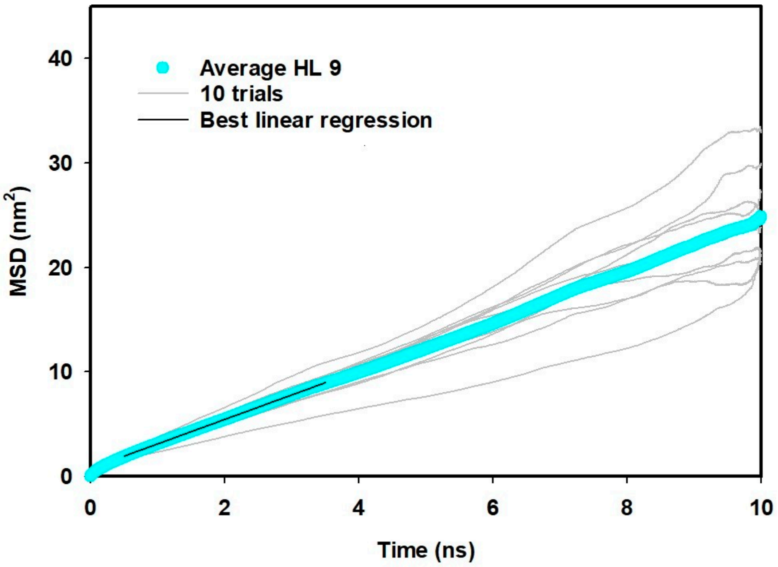

Figure S22: MSD plot and the trend line at the HL 9 for H_2O ions in the presence of QCS (C) of AEM.

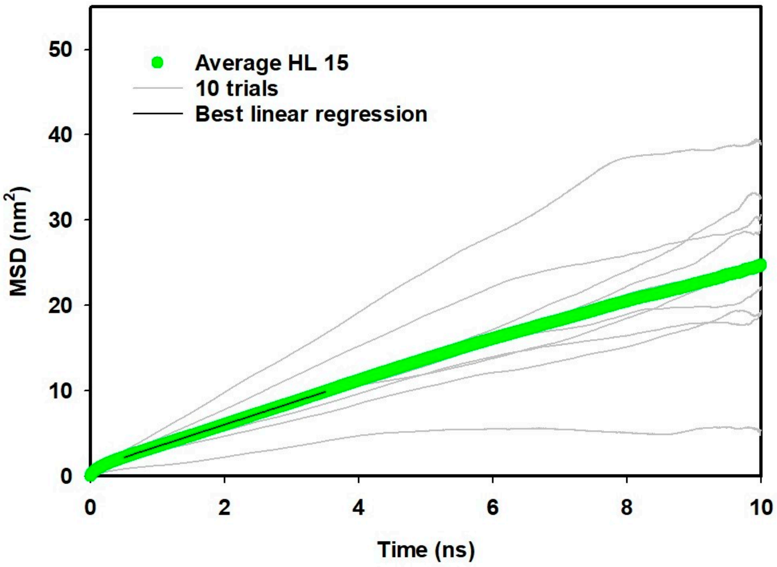

Figure S23: MSD plot and the trend line at the HL 15 for H_2O ions in the presence of QCS (C) of AEM.

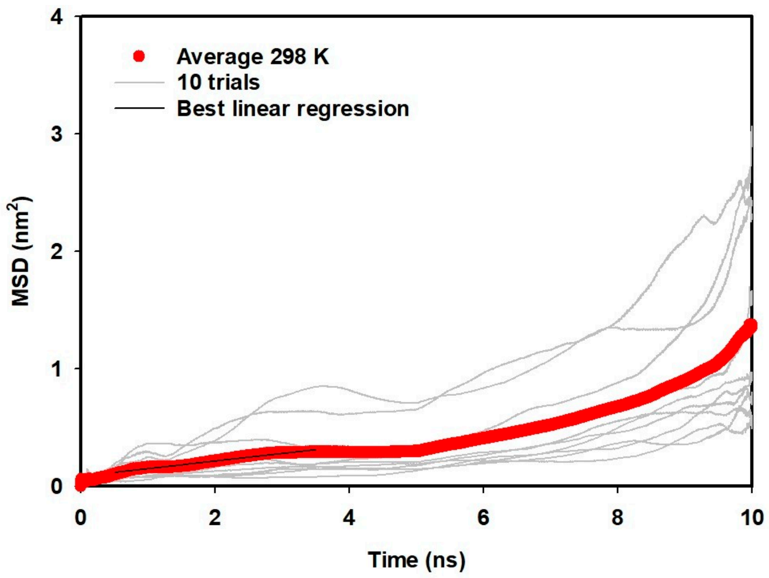

Figure S24: MSD plot and the trend line at the HL 3 for OH^- ions in the presence of QCS (A) of AEM at 298 K.

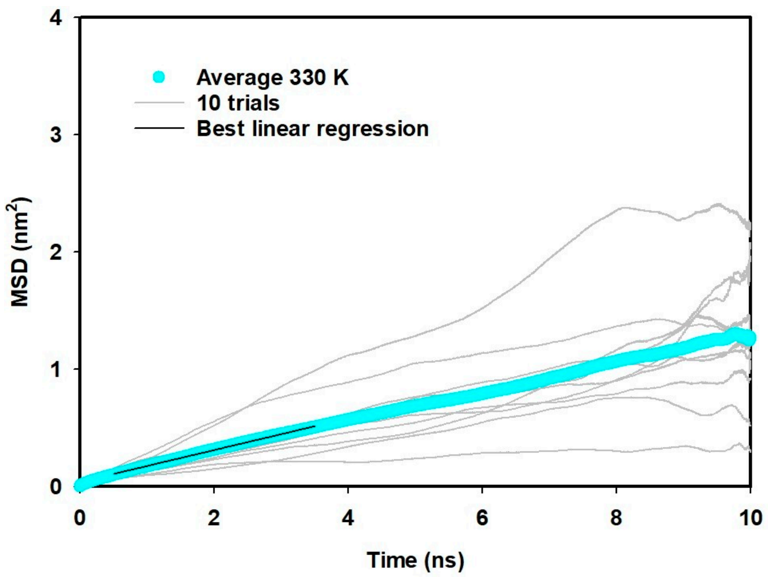

Figure S25: MSD plot and the trend line at the HL 3 for OH^- ions in the presence of QCS (A) of AEM at the 330 K.

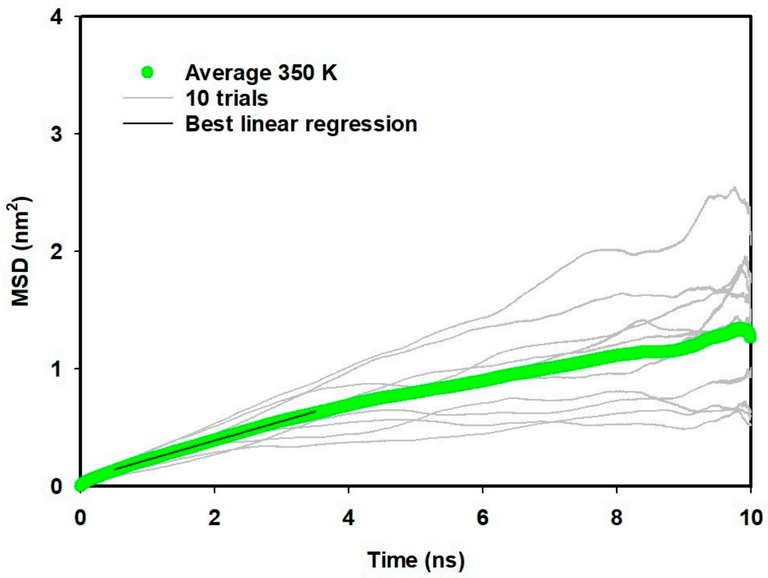

Figure S26: MSD plot and the trend line at the HL 3 for OH^- ions in the presence of QCS (A) of AEM at the 350 K.

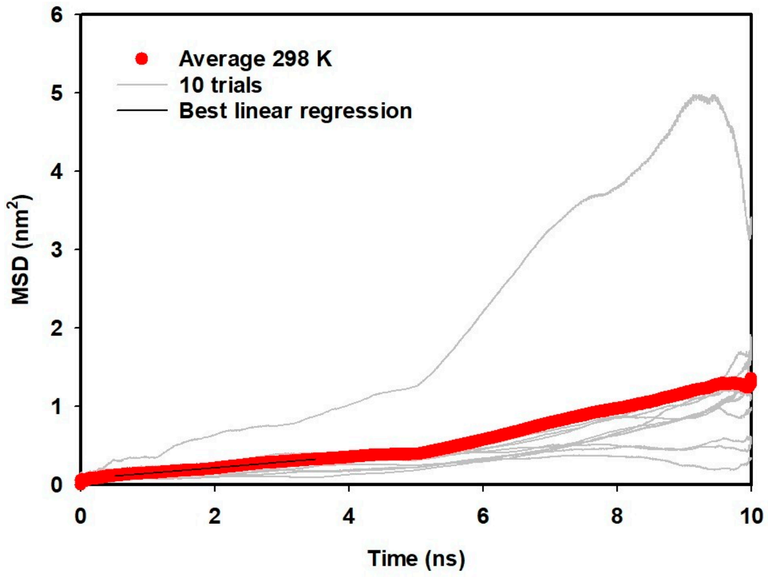

Figure S27: MSD plot and the trend line at the HL 3 for OH^- ions in the presence of QCS (B) of AEM at the 298 K.

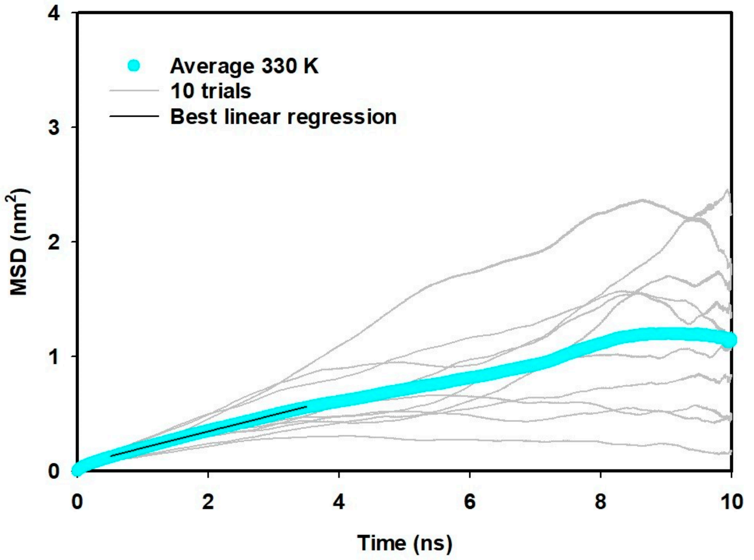

Figure S28: MSD plot and the trend line at the HL 3 for OH^- ions in the presence of QCS (B) of AEM at the 330 K.

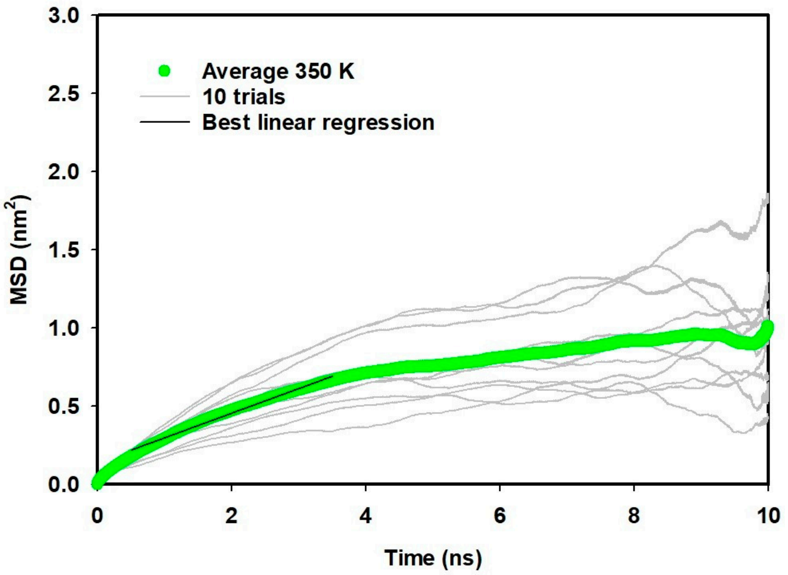

Figure S29: MSD plot and the trend line at the HL 3 for OH^- ions in the presence of QCS (B) of AEM at the 350 K.

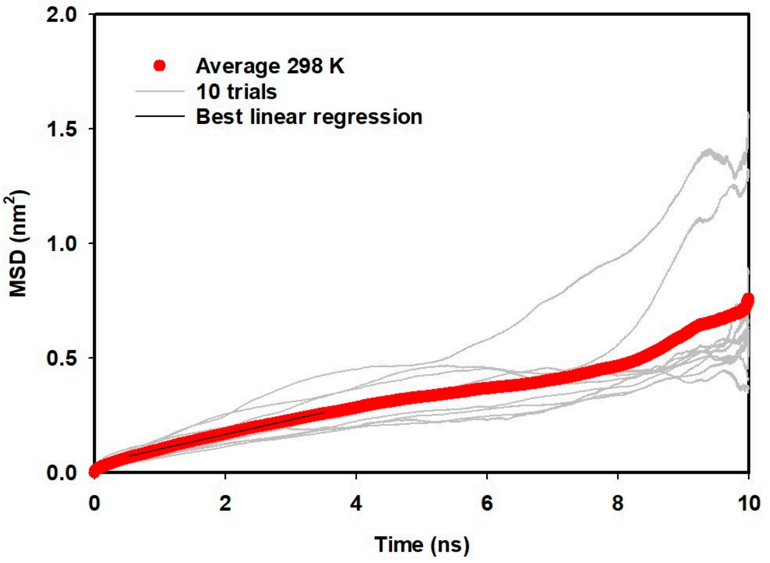

Figure S30: MSD plot and the trend line at the HL 3 for OH^- ions in the presence of QCS (C) of AEM at the 298 K.

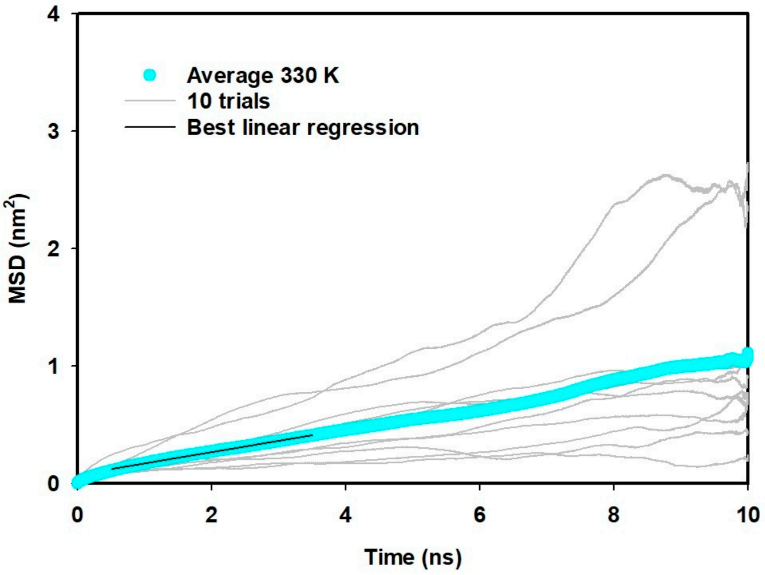

Figure S31: MSD plot and the trend line at the HL 3 for OH^- ions in the presence of QCS (C) of AEM at the 330 K.

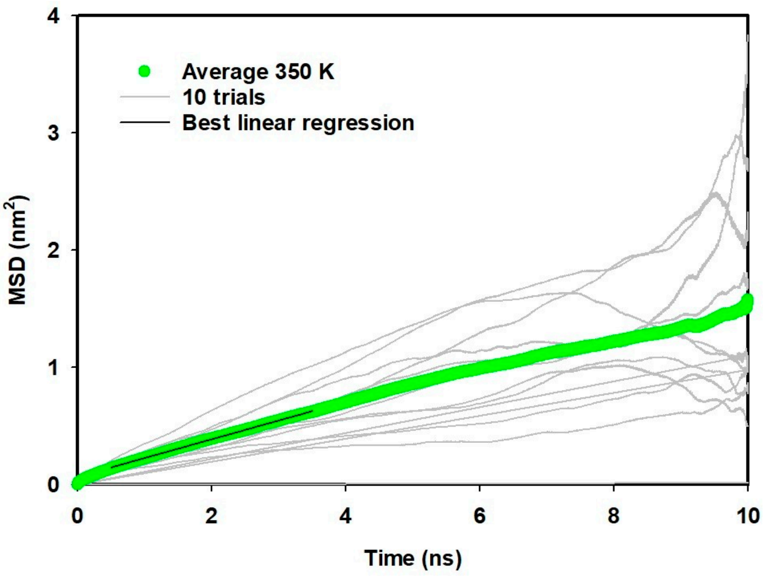

Figure S32: MSD plot and the trend line at the HL 3 for OH^- ions in the presence of QCS (C) of AEM at the 350 K.

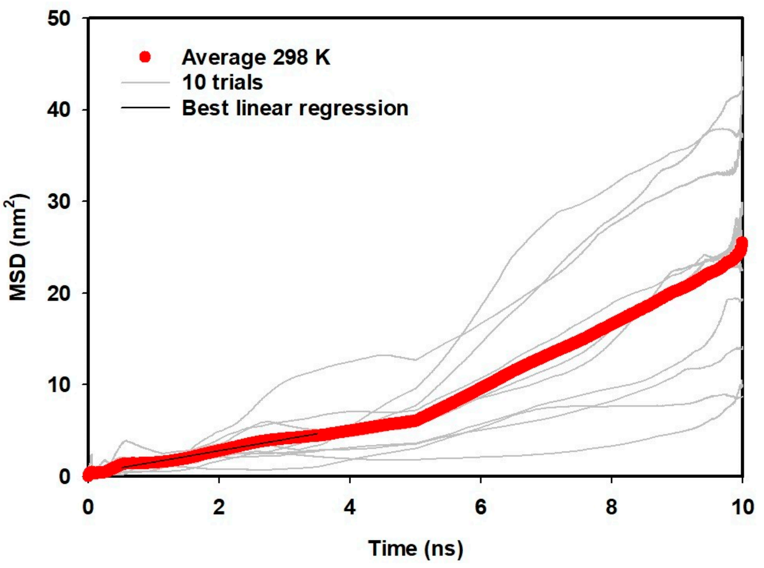

Figure S33: MSD plot and the trend line at the HL 3 for H_2O ions in the presence of QCS (A) of AEM at the 298 K.

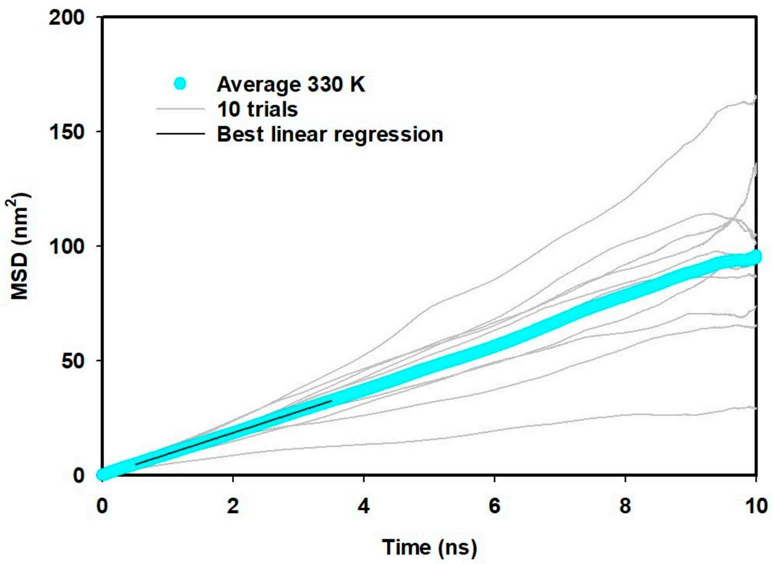

Figure S34: MSD plot and the trend line at the HL 3 for H_2O ions in the presence of QCS (A) of AEM at the 330 K.

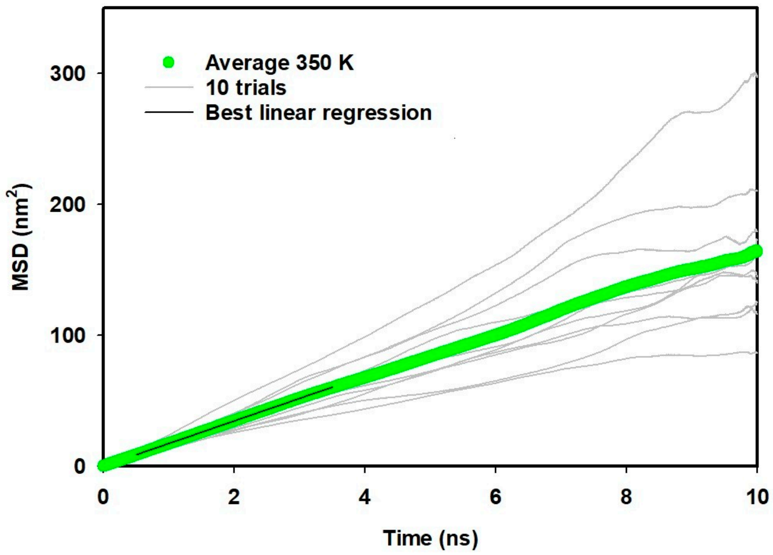

Figure S35: MSD plot and the trend line at the HL 3 for H_2O ions in the presence of QCS (A) of AEM at the 350 K.

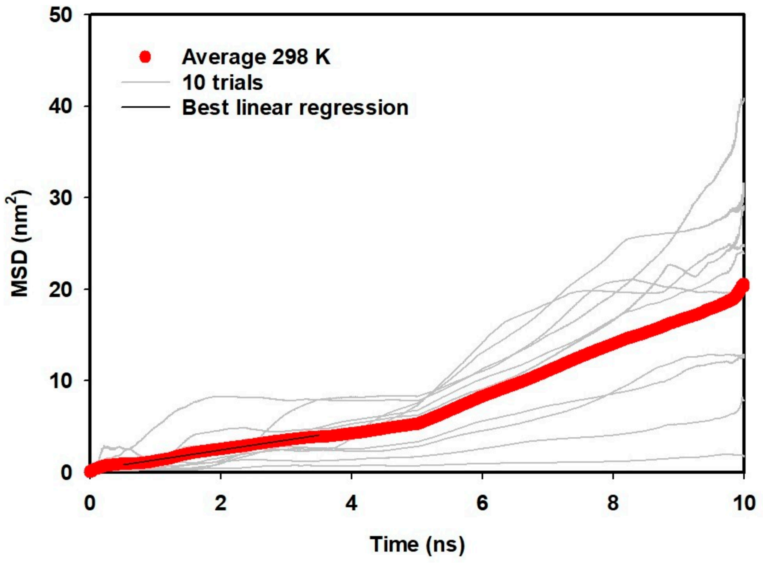

Figure S36: MSD plot and the trend line at the HL 3 for H_2O ions in the presence of QCS (B) of AEM at the 298 K.

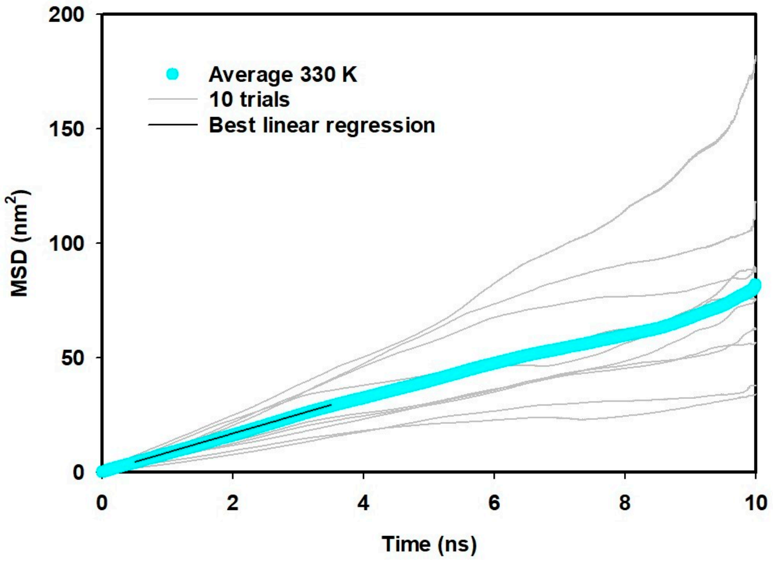

Figure S37: MSD plot and the trend line at the HL 3 for H_2O ions in the presence of QCS (B) of AEM at the 330 K.

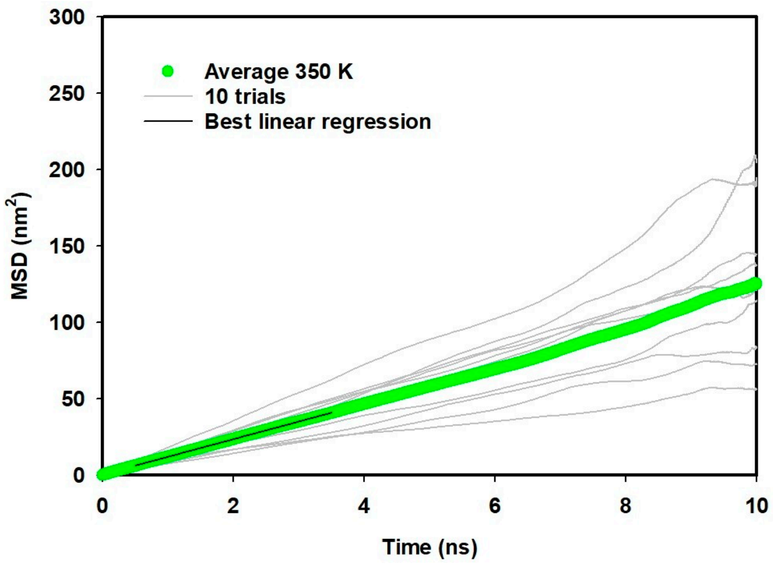

Figure S38: MSD plot and the trend line at the HL 3 for H_2O ions in the presence of QCS (B) of AEM at the 350 K.

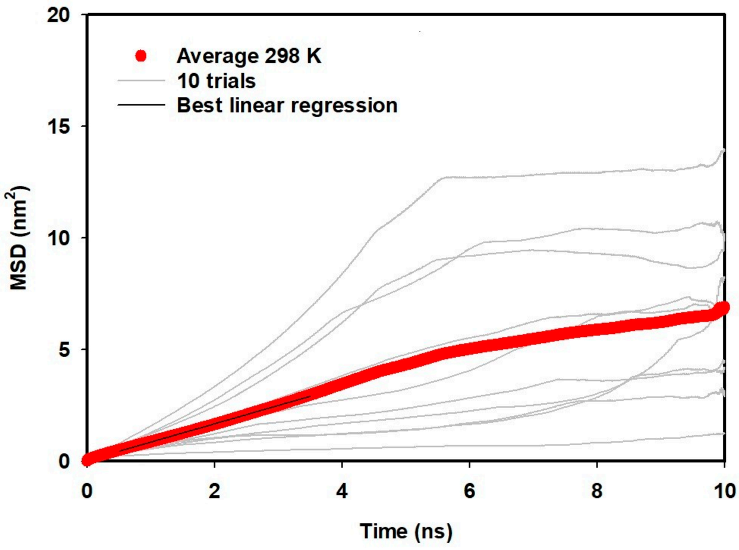

Figure S39: MSD plot and the trend line at the HL 3 for H_2O ions in the presence of QCS (C) of AEM at the 298 K.

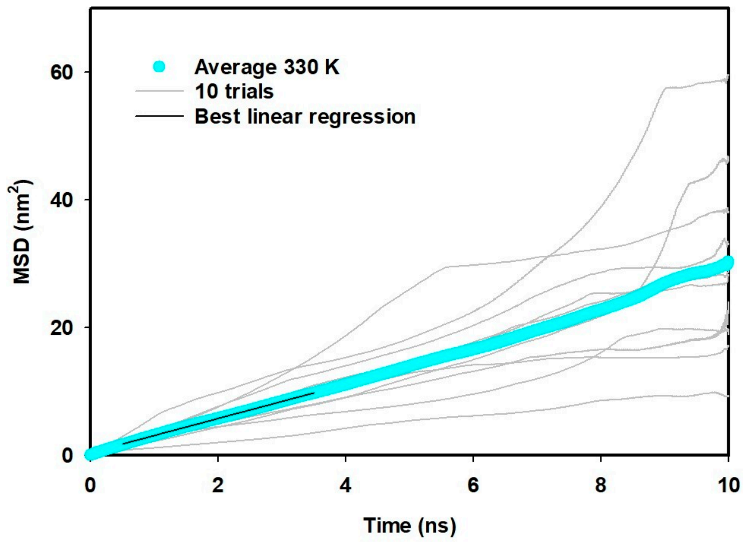

Figure S40: MSD plot and the trend line at the HL 3 for H_2O ions in the presence of QCS (C) of AEM at the 330 K.

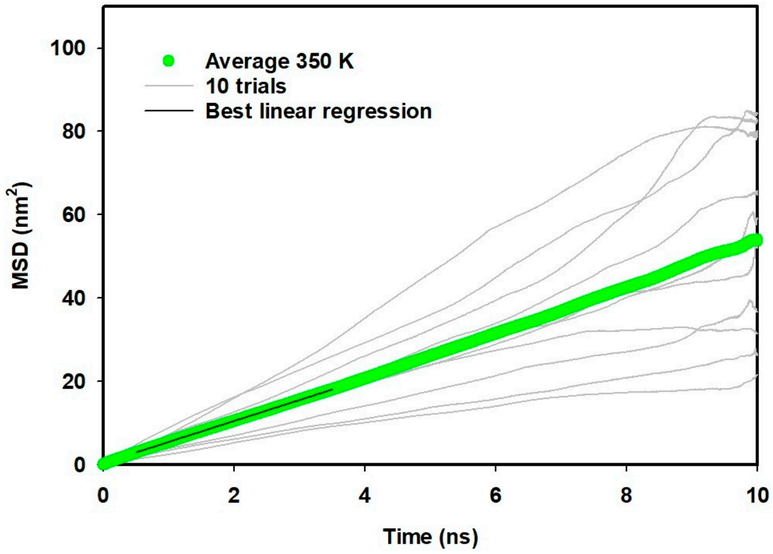

Figure S41: MSD plot and the trend line at the HL 3 for H_2O ions in the presence of QCS (C) of AEM at 350 K.
